# Screening Cobalt-based Catalysts on Multicomponent CdSe@CdS Nanorods for Photocatalytic Hydrogen Evolution in Aqueous Media

**DOI:** 10.1021/acsanm.4c01645

**Published:** 2024-06-18

**Authors:** Marcel Boecker, Sarah Lander, Riccarda Müller, Anna-Laurine Gaus, Christof Neumann, Julia Moser, Mathias Micheel, Andrey Turchanin, Max von Delius, Christopher V. Synatschke, Kerstin Leopold, Maria Wächtler, Tanja Weil

**Affiliations:** † Department for Synthesis of Macromolecules, Max Planck Institute for Polymer Research, Mainz 55128, Germany; ‡ Department of Chemistry and State Research Center OPTIMAS, RPTU Kaiserslautern-Landau, Kaiserslautern 67663, Germany; § Institute of Analytical and Bioanalytical Chemistry, University Ulm, Ulm 89081, Germany; ∥ Institute of Organic Chemistry I, University Ulm, Ulm 89081, Germany; ⊥ Institute of Physical Chemistry, Friedrich Schiller University Jena, Jena 07743, Germany; # Abbe Center of Photonics (ACP), Jena 07745, Germany

**Keywords:** photocatalysis, polydopamine, CdS nanorods, cobalt catalyst, photocatalytic system

## Abstract

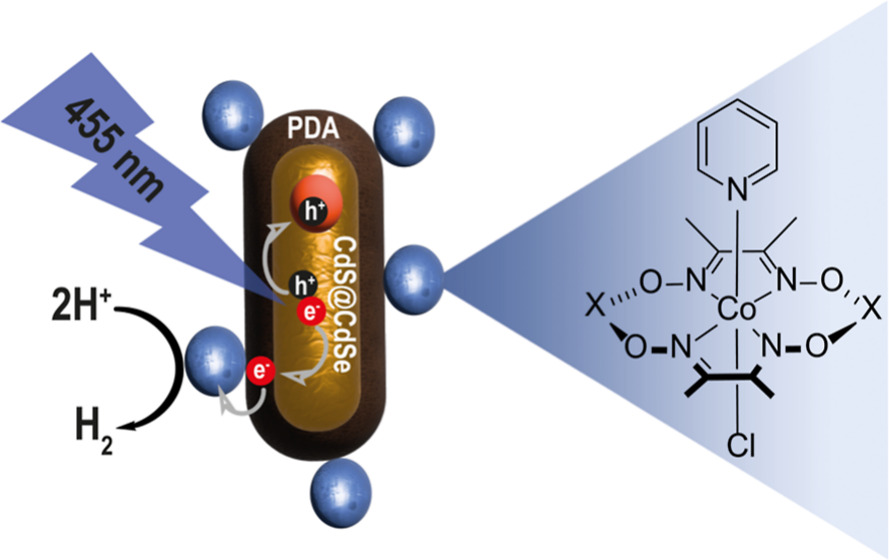

We present CdSe@CdS nanorods coated with a redox-active polydopamine (PDA) layer functionalized with cobaloxime-derived photocatalysts for efficient solar-driven hydrogen evolution in aqueous environments. The PDA-coating provides reactive groups for the functionalization of the nanorods with different molecular catalysts, facilitates charge separation and transfer of electrons from the excited photosensitizer to the catalyst, and reduces photo-oxidation of the photosensitizer. X-ray photoelectron spectroscopy (XPS) confirms the successful functionalization of the nanorods with cobalt-based catalysts, whereas the catalyst loading per nanorod is quantified by total reflection X-ray fluorescence spectrometry (TXRF). A systematic comparison of different types of cobalt-based catalysts was carried out, and their respective performance was analyzed in terms of the number of nanorods and the amount of catalyst in each sample [turnover number, (TON)]. This study shows that the performance of these multicomponent photocatalysts depends strongly on the catalyst loading and less on the specific structure of the molecular catalyst. Lower catalyst loading is advantageous for increasing the TON because the catalysts compete for a limited number of charge carriers at the nanoparticle surface. Therefore, increasing the catalyst loading relative to the absolute amount of hydrogen produced does not lead to a steady increase in the photocatalytic activity. In our work, we provide insights into how the performance of a multicomponent photocatalytic system is determined by the intricate interplay of its components. We identify the stable attachment of the catalyst and the ratio between the catalyst and photosensitizer as critical parameters that must be fine-tuned for optimal performance.

## Introduction

Hydrogen is considered a key part to future CO_2_-neutral production of energy and raw materials for the (chemical) industry.^1,2^ It has the highest known energy density of any fuel (120 MJ kg^–1^), more than twice the calorific value of most conventional fuels,^2^ and it is nontoxic and environmentally friendly as its combustion product is water. However, the problem with today’s hydrogen production is that 98% derives from fossil sources and its production cannot be considered environmentally friendly.^3^ Therefore, a “holy grail” of sustainable “green” hydrogen production methods is water splitting powered by sunlight.^1^

Solar-driven water splitting systems based on semiconductors have shown remarkable potential for photocatalytic hydrogen production^4,5^ and typically involve four key processes: Light absorption, charge carrier separation, accumulation of redox equivalents, and catalytic reaction.^6,7^ For the system to function properly, the band-level potentials of the semiconducting materials used must match the redox potentials for both the proton-reduction and water-oxidation half-reactions. This results in a theoretical minimum band gap of 1.23 V, which in reality increases to 2 to 2.4 eV due to kinetic overpotentials and energy losses during the process.^8,9^ Cadmium sulfide is an excellent semiconducting material because it meets the necessary band gap requirements and can absorb solar radiation in the visible spectral range.^8,10^

In semiconductor-based systems, nanostructuring has proven to be an important factor in minimizing distances for charge carrier migration and thus preventing recombination before it is successfully transferred to reactive sites at the surface. Colloidal semiconductor nanocrystals have great potential in this respect. They are tunable in size, shape, and composition through small adjustments in the synthetic parameters, which allows exquisite control over their photochemical properties.^11^ Moreover, the choice of surface ligands enables control of the surface chemistry and electronic structure of the nanocrystals as well as their dispersion in various solvents. In this study, we used a special type of heterostructure consisting of a CdSe core embedded in a CdS rod to achieve spatial separation of the redox half-reaction sites.^12,13^ In CdSe@CdS nanorods (NRs) with quasi-type II band alignment, hole localization takes place in the CdSe seed, while electrons are delocalized over the entire rod and rapidly migrate to the cocatalyst on ps-time scales, thus increasing catalytic turnover.^14,15^ CdSe@CdS reveals high quantum efficiencies for light-driven hydrogen generation in an aqueous solution in combination with cocatalysts such as metal nanoparticles,^14^ redox-active enzymes,^16^ or transition metal complexes.^17^ However, the long-term performance of these systems is limited by photooxidation processes, induced by unquenched holes in the NR core.^18,19^ This degradation needs to be prevented by establishing efficient hole-extraction pathways, i.e., via sacrificial electron donors, coupling with OER, or coupling with alternative oxidation reactions, creating valuable products.^19^

Bioinspired redox active polymers such as melanin-mimicking bioinspired polydopamine (PDA) could improve photocatalytic efficiency in several photocatalytic systems.^20,21^ PDA is formed by the autoxidation of dopamine, resulting in multifunctional coatings.^22^ It provides adhesive and protective layers^23^ and strong coordination bonds preventing ligand loss caused by oxidation.^24,25^ Moreover, electron transport to the reaction centers can also be achieved by the catechol/quinone moieties of the PDA layer similar to the natural photosystem II.^26^ The improved photocurrent and photocatalytic performance of, e.g., CdS/PDA/TiO_2_ hybrid nanoparticles^24^ is either due to the availability of electron-accepting groups in PDA that support electron transport^26^ or by direct electron tunneling in the case of very thin layers.^23^ Additionally, PDA also offers functionalities for surface functionalization,^27,28^ for example, PDA-coated CdSe@CdS nanorods were functionalized with the water-insoluble Rh-based catalyst, and the photocatalytic reduction of NAD^+^ to NADH in pure aqueous solution was achieved successfully.^29^

Cobaloximes as potential cocatalysts have revealed unique advantages.^30^ These cobalt-based molecular catalysts have shown a high proficiency for proton reduction, closely mimicking natural enzymes.^31^ In particular, cobaloximes, which are mainly used in homogeneous systems either in simple mixtures with photosensitizers^32^ or in molecular sensitizer-catalyst diads,^33^ ensure uniform distribution and consistency in the reaction environment, providing a controlled platform for hydrogen evolution.^30,34^ CdS-based semiconductor nanocrystals are interesting alternatives to common molecular sensitizers, and integrating cobaloxime catalysts with CdS semiconductor-based sensitizers could significantly advance the development of efficient and sustainable hydrogen production methodologies but presents several challenges, e.g., interfacing semiconductor nanocrystal-based sensitizers with molecular catalysts, i.e., linking catalyst and nanocrystal while sustaining dispersibility of the particles and balancing charge separation versus recombination. Various strategies based on electrostatic interactions^34^ or covalent bonding^35,36^ can be pursued. In addition, one goal would be to develop systems that can be used exclusively in a water-based environment without the need for cosolvents to ensure the dispersibility of the catalyst, as is the case with a simple mixture of sensitizer and catalyst.

**Scheme 1:**
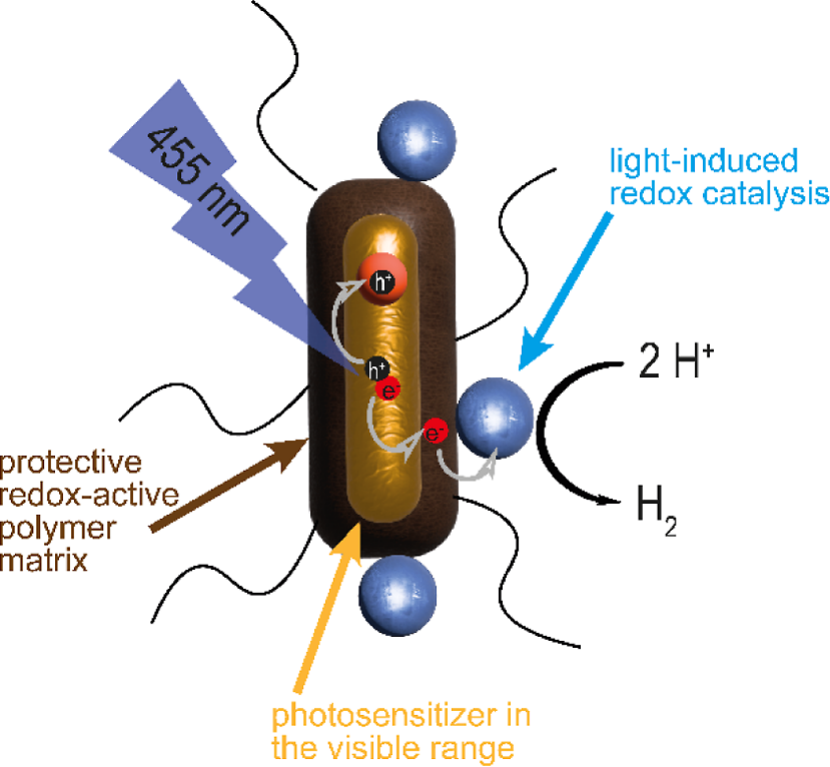
Schematic Drawing of the Synthesized Photocatalytic System, Consisting of CdSe@CdS Nanorods (NRs) as Photosensitizers, PDA as a Multifunctional Matrix, and Molecular Cobalt-based Catalysts, with the Expected Charge Transfer Processes during the Photocatalytic Reaction

In the present study, we combined PDA-coated CdSe@CdS nanorods with a series of cobaloxime catalysts with proven catalytic activity for HER.^37−39^ This approach overcomes the limitations of the Co-catalysts, such as their very low water-solubility, and demonstrates hydrogen evolution in aqueous media.^31,40−42^ Our results pave the way for developing highly efficient and stable photocatalytic systems for sustainable hydrogen production. By integrating PDA-coated CdSe@CdS nanorods with cobaloxime catalysts, we address the challenges of both charge separation and efficient HER catalysis. Immobilizing cobaloxime catalysts onto the nanorod surface enables their utilization in aqueous media, and the integration of cobaloxime catalysts with PDA-coated nanorods provides a stable platform for catalyst immobilization, enhancing both the catalyst stability and recyclability. Additionally, the spatial arrangement of components within the heterostructure facilitates efficient charge transfer and separation, leading to enhanced catalytic activity. Based on observations in our previous study,^29^ we can propose a mechanism that involves the transfer of electrons after excitation from conduction band states to the polydopamine, which is potentially capable of serving as electron relay shuttling the charge carriers to the Co catalyst reaction center (Scheme 1). This innovative approach opens avenues for scalable, environmentally friendly hydrogen generation in an all-aqueous environment, marking significant progress toward realizing the potential of solar-driven water splitting as a clean energy solution.

## Results and Discussion

The photocatalytic system consists of CdSe@CdS nanorods (NRs) as photosensitizers, PDA as a multifunctional matrix, and molecular cobalt-based catalysts that were associated with the PDA layer (Scheme 1).

**Scheme 2:**
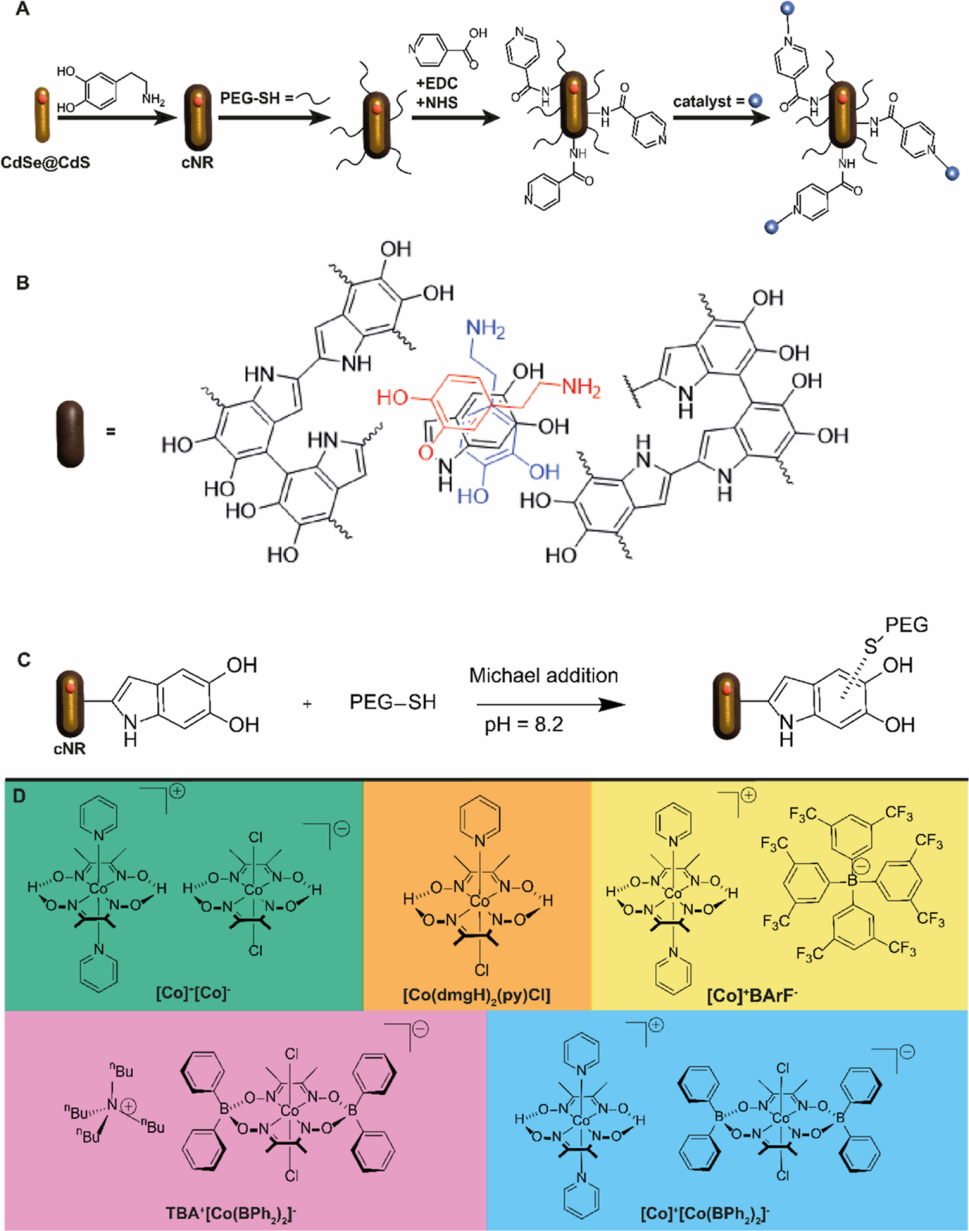
Synthesis-Scheme for the Photocatalytic System Based on CdSe@CdS Nanorods Coated with PDA to Yield cNR, Followed by Functionalization with PEG-SH via Michael Addition and Isonicotinic Acid via Amid Formation and Catalyst Association to Yield cNR-PEG-Cat (A), a Schematic Illustration of the PDA Structure (B), Reaction Scheme of the Functionalization with PEG via Michael Addition (C), and Structures of the Five Different Cobalt-based Catalysts (D)

A simplified reaction scheme for the design of this multicomponent photocatalytic system, including the PDA structure, the functionalization via Michael addition and the selected Co-based catalysts, are shown in Scheme 2.^37^ The CdSe@CdS nanorods (length: 43.8 ± 5.8 nm, width: 4.8 ± 0.4 nm, Figure S1) were synthesized by a seeded growth approach (CdSe core diameter: 2.0 nm) and further transferred into aqueous solution by ligand exchange of the surface ligands of the as-synthesized nanocrystals with mercaptoundecanoic acid.^14^ In the next step, the NRs were coated with PDA via autoxidation of dopamine in alkaline tris(hydroxymethyl)aminomethane (TRIS) buffer (0.1 M, pH = 8.5), resulting in PDA-coated NR (cNR) with a < 5 nm thin PDA shell,^29^ subsequent functionalization of the NRs was accomplished with polyethylene glycol via Michael addition to improve dispersion in aqueous media as well as by condensation of isonicotinic acid for binding the Co-catalysts. Co-based catalysts were synthesized following a previously published protocol^37^ and additionally a commercially available cobaloxime ([Co(dmgH)_2_(py)Cl]) catalyst was used in this study for comparison (for the respective structures see Scheme 2D). In previous work on this cobaloxime complex salts, the nature of counteranions, which are either cobalt-based or innocent (e.g., BArF), was found to have an effect on the stability of catalysts [turnover number (TON)]^37^ as well as solubility.^39^ The effect of counteranions on the immobilization efficiency, which plays a role in this work, is a new research question in the context of this compound class. In the last step, the catalysts were attached to the cNR-PEGs by mixing the dissolved catalysts (in acetonitrile) and the cNR-PEGs in phosphate buffer (0.1 M, pH = 7). Purification was done by 3 times filtering through a centrifuge filter at 4000*g* for 5 min, and the remaining photocatalytic systems were redispersed in Milli-Q water to give the different cNR-PPEG-Cat, respectively, cNR-PEG-[Co]^+^[Co]^−^(I), cNR-PEG-[Co(dmgH)_2_(py)Cl] (II), cNR-PEG-[Co]^+^BArF^–^ (III), cNR-PEG-TBA^+^[Co(BPh_2_)_2_]^−^ (IV), and cNR-PEG-[Co]^+^[Co(BPh_2_)_2_]^−^ (V). For further details on the synthesis, see the Supporting Information under Materials and Synthesis chapter.

We have studied the formation of the PDA shell on the NRs previously.^29^ The thus formed coated cNR showed a broadened absorption spectrum compared to bare CdSe@CdS NRs, and the PDA shell nearly completely quenched the photoluminescence of the NRs due to efficient charge transfer from the photoexcited NRs to the PDA shell (Figure S2).^29^ Furthermore, EELS mapping of the cNR was measured, showing the presence of Cd and S only in the nanorod structure (Figure S3). For Se, the intensity is very low, resulting in a poor signal-to-noise ratio, which can be explained due to the fact that Se is only present inside the small core in the nanorod structure. From the mapping of the PDA elements C, N, and O a homogeneous distribution can be observed, additionally proving the homogeneous coating of the NR.

**Figure 1:**
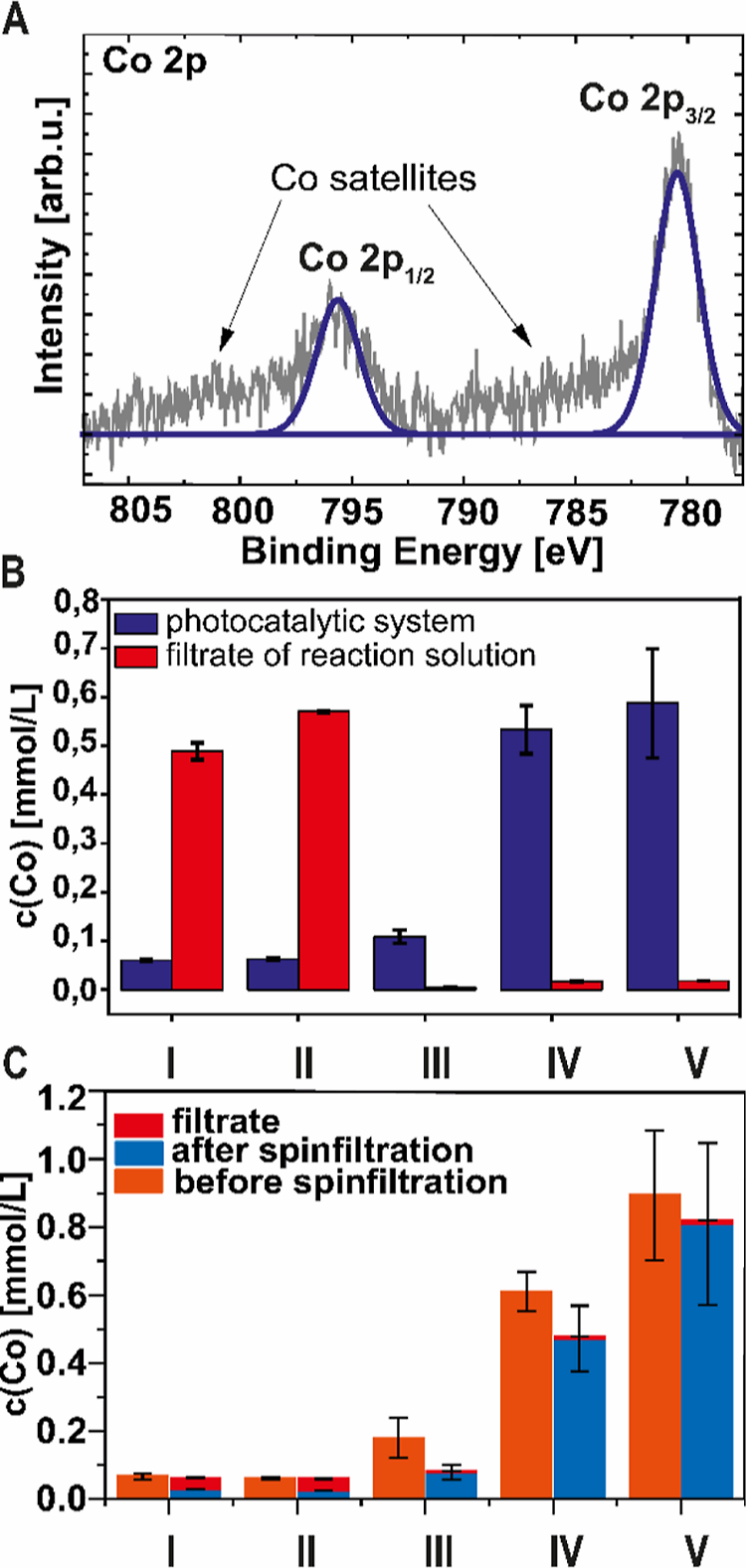
High-resolution Co 2p XP spectra of photocatalytic system IV (A), cobalt concentration of the five different cNR-PEG-Cat and the reaction solution filtrate measured by TXRF (B), and cobalt concentration after a week of storage in Milli-Q water before and after filtration and in the filtrate measured by TXRF (C).

X-ray photoelectron spectroscopy (XPS) was used to determine the successful functionalization of the NRs. The cNRs show the typical PDA peaks in the C 1s, O 1s, and N 1s spectra (Figure S4), respectively.^29^ This is reflected in the dominant C–C/C–H (284.6 eV) feature in the C 1s spectrum, which is accompanied by two shoulders assigned to the C–N/C–O (286.1 eV) and C=O/COOH (288.2 eV) bonds. Furthermore, the S 2p spectrum shows two species: one of the CdS nanorod and one sulfur-organic bond, indicating PDA binding to the NR. The latter assignment is supported by the O 1s and N 1s spectra. Polydopamine-coated nanorods were functionalized only with isonicotinic acid and without the PEGylation to determine a successful functionalization in the high-resolution XP spectra. Due to the functionalization, the C 1s peaks assigned to C–N (285.8 eV) and COOH (288.2 eV) increase. Similarly, the double bonds of carbon and oxygen in the O 1s spectrum (531.5 eV) compared to the cNR indicate successful functionalization (Figure S5). The same applies to the cNR-PEG. The amount of carbon and oxygen single (286.1 eV) and double bonds (288.2 eV) in the C 1s spectrum is increased, therefore indicating a successful functionalization with PEG (Figure S6). The cNR-PEG with isonicotinic acid shows the highest amount of the different oxygen species in the C 1s and the O 1s (Figure S7) even compared to the single functionalized nanorods (cNR-PEG or cNR with isonicotinic acid), indicating the successful successive functionalization of the cNR with PEG and isonicotinic acid. Furthermore, after conjugation of the cobalt-based catalysts, a Co signal was detected in XPS (Figures 1A and S8–S12) in all five samples, which further indicates successful functionalization of the NRs with the Co-catalysts. The Co 2p signals show the characteristic doublet accompanied by broad satellite features, which are typical for transition metal spectra. Due to the low cross-section of boron and the present phosphate signal in the same binding energy region (P 2s) caused by the remains of the phosphate buffer salts from synthesis, no B 1s signal could be measured. However, for the photocatalytic system III, a fluorine signal (F 1s) was detected in the XP spectra (Figure S12), indicating that the counterion is still present in this catalytic system.

To quantify the functionalization of the photocatalytic system, the amount of the elements of the catalyst (Co) and the photosensitizer (Cd, Se, and S) were determined by total-reflection X-ray fluorescence spectrometry (TXRF). The values of the Cd and Co content are listed for all photocatalytic systems in Table S2 (Supporting Information). The highest amount of immobilized Co-catalysts was detected for the photocatalytic systems IV (0.53 ± 0.05 mmol/L) and V (0.6 ± 0.1 mmol/L) (Figure 1B), while 10-times lower Co-concentrations were found for the photocatalytic systems II (0.063 ± 0.003 mmol/L) and I (0.060 ± 0.002 mmol/L). This trend in concentration is reversed when the filtrate of the catalyst association solution is analyzed (Figure 1B), as samples with a low coupling efficiency show high concentrations of free cobalt. One interesting observation is that the photocatalytic system V with the cobalt double salt [Co]^+^[Co(BPh_2_)_2_]^−^ as the catalyst showed the highest Co concentration, whereas (I, with [Co]^+^ [Co]^−^) showed the lowest Co concentration at the NRs. At least for these two examples, the double salt nature of the catalysts does not lead to consistently high levels of catalyst association. At first glance, the BPh_2_ bridge seems to further increase the immobilization efficiency, perhaps by additional π–π* or van der Waals interactions between the ligand and the PDA coating.

To evaluate the stability of the attached catalysts with time, we kept the multicomponent photocatalyst systems in solution under atmospheric conditions for 1 week. The Co concentration was determined in the stored solution (without purification), after spin-filtration to eliminate free cobalt complexes from the solution, and in the filtrate (Figure 1C). Compared to the initial samples, no changes were observed for the nonpurified solution, and the Co content decreased only marginally for all catalysts after filtration. Also, only very small amounts of Co were detected in the filtrate. Furthermore, the stability of the NRs during the functionalization and purification procedure were evaluated, and no Cd, Se, or S were detected in the filtrate of the reaction solution. These results demonstrate the robustness of the photocatalytic systems, and almost no catalyst detachment was observed after 1 week of storage.

We then estimated the amount of catalyst per nanorod. First, we quantified the amount of nanorods in each sample by determining the Cd concentration, which was consistently around 2 mmol/L for all samples, except for photocatalytic system V (3.6 mmol/L), indicating a slightly larger number of NRs in this sample (Figure S13). By using the measured Cd concentration, the NR size (as determined by SEM in Figure S1), and the wurtzite crystal structure of the NRs, the approximate number of nanorods present in the photocatalytic solution was calculated (see the Instruments and Techniques section and Table S2 in the Supporting Information). Consequently, each photocatalytic experiment involved approximately 40 nmol rods except for the photocatalytic system V which involved 70 nmol. However, the same catalyst association trend in the molar Co/Cd ratio can still be observed (Figure S14), and the above statement, that the double salt nature does not lead to higher catalyst association, remains valid.

**Figure 2:**
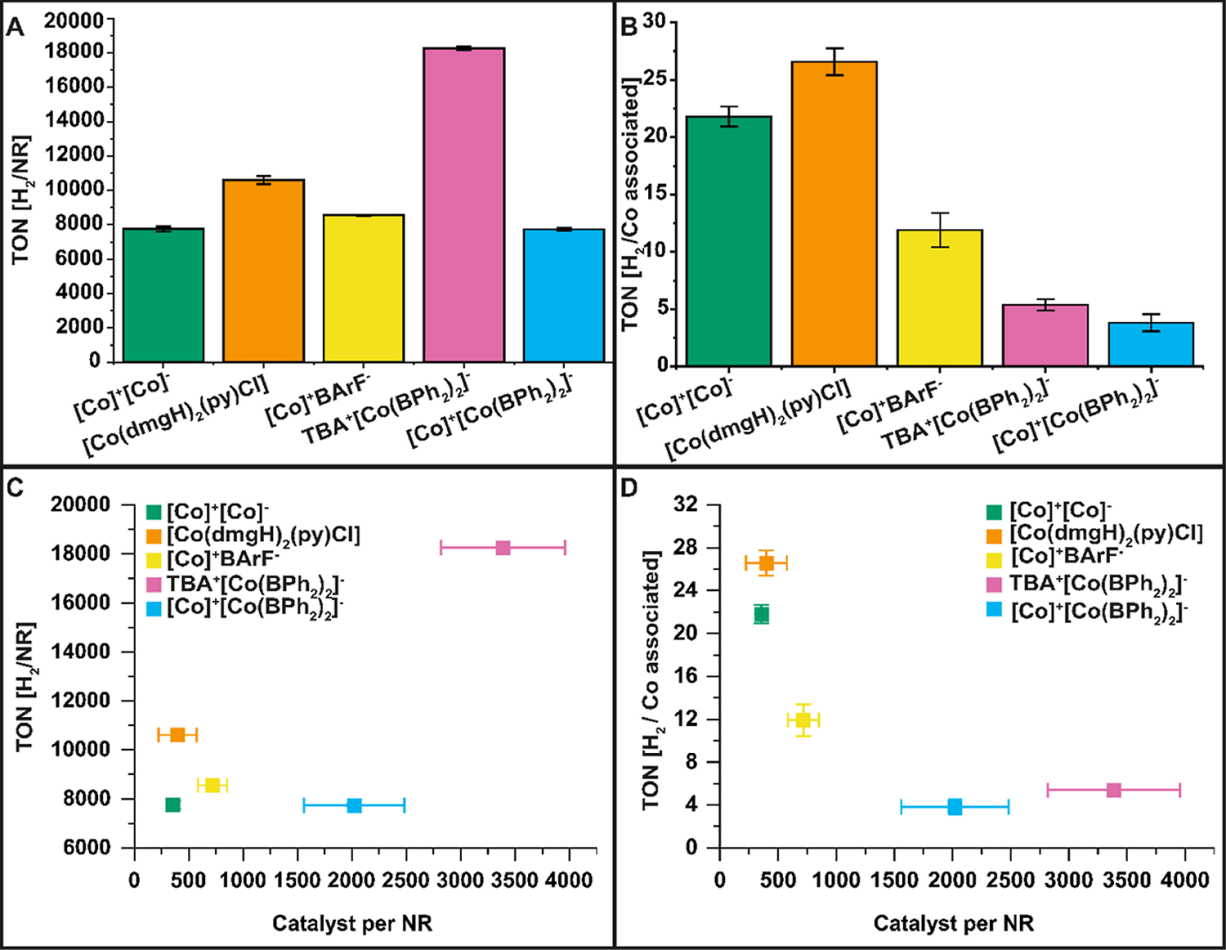
Measured amounts of photocatalytically produced hydrogen normalized by the number of NR (photosensitizer, A) and normalized by the amount of catalyst (TON, B). The determined hydrogen per NR against the amount of catalyst per NR (C). The determined TON against the amount of catalyst per NR (D).

Combining the Co concentration and the amount of NR, we then determined the average amount of catalyst per rod (Figure 2C,D and Table S2). As seen previously for the concentration of Co, the photocatalytic system I has one with the lowest amount of catalyst per rod with 360 ± 40 catalytic centers per rod. On the other hand, in the case of IV, almost 10 times more catalysts have been immobilized on average per rod, yielding 3400 ± 600 catalytic centers per rod. While the photocatalytic systems II and III have similar amounts of associated catalyst compared to I, only the photocatalytic system V shows a significantly higher amount with 2021 ± 461 catalytic centers/rod. Taking the different catalyst structures into account, it seems that the catalysts that have phenyl groups in the ligand structure have an advantage for catalyst association to the cNR-PEG, independent of the counterion or double salt nature.

After the careful and in-depth characterization of the complete photocatalytic system, photocatalytic hydrogen evolution measurements were performed (three runs for each system) by dispersing the cNR-PEG-Cats in an ascorbic acid buffer (pH = 5, 0.1 M), followed by irradiation for 6 h with a 455 nm LED. The amount of produced hydrogen was determined through headspace gas chromatography (GC). For the cNR without any catalyst attached, there was no hydrogen detected by GC. For all values, the error of the hydrogen amount was determined by standard deviation, and the errors of the two different TON values were calculated by error propagation, and all values are listed in Table S2. The photocatalytic system IV showed the highest hydrogen amount per NR (photosensitizer, 18,252 ± 105 H_2_/rod), whereas both cobalt double salt materials (V and I) generated the lowest amount of hydrogen per rod (7741 ± 79 H_2_/rod and 7755 ± 163 H_2_/rod, respectively). Since the intrinsic photocatalytic HER activity of all molecular catalysts is similar within a factor of the associated catalysts associated with each rod, these differences in photogenerated hydrogen should relate to the design of the photocatalytic system. As described above, the amount of attached catalyst varies significantly between the different photocatalytic systems. Thus, we calculated the TON per catalyst. This approach also allowed us to compare our results with a previous study on these catalyst complexes in a homogeneous photocatalysis, which used a ruthenium complex as a photosensitizer, to see if the catalysts lose reactivity due to immobilization.^37^ According to the previous publication on those catalysts in solution, we assumed that both cobalt centers are active in the catalysis for the TON calculation of the Co double salts.^37^ The observed TONs vary between 4 and 27 mol H_2_/mol catalyst and are thus in a similar range as previously reported for this series of catalysts in a purely molecular scheme with a molecular Ruthenium-based photosensitizer (TONs between 35 and 65)^37^ and with similar cobaloximes electrostatic adsorbed at CdS photosensitizers (TOF between 3 and 10 for 1 h irradiation)^34^ even though the catalytic conditions (e.g., irradiation intensity and duration or solvents) will vary for the reported literature values to ours. Therefore, the catalysts do not dramatically lose activity due to association with our cNR-PEG system, while the system can easily be recovered by spin filtration, in contrast to homogeneous catalysis. We observe that the TONs in dependence of catalyst loading (Figure 2D) are highest for the samples with the lowest catalyst loading. This behavior can be rationalized with a competition of the catalysts for charge carriers: each catalyst needs to accumulate two electrons to drive the reduction of proton to molecular hydrogen, which is less likely to occur when catalyst surface coverage increases. This effect has been reported for metal particle-functionalized colloidal nanorods^43,44^ and potentially also plays a role in molecularly functionalized systems.^36^

Our observations indicate that the photocatalytic efficiency of our catalytic systems is not solely determined by the intrinsic nature of the catalyst but is significantly influenced by the overall molecular architecture of the fully assembled system as well as by the catalyst loading, which may result in different performance trends within our series compared to observations in a mixture with molecular sensitizers.^37^

For instance, we found that the BPh_2_ bridge of the ligand enhances solution photocatalytic HER activity due to higher chemical stability,^37^ while the opposite effect is observed in the cNR-PEG-Cat materials presented in this study. This trend is attributed to the unique interaction between the sensitizer and catalyst in our system, resulting in a higher concentration and a higher number of catalysts binding to the particle surface. These findings have important implications for the design of optimized photocatalytic hybrid molecular-polymer-semiconductor nanoparticle systems. Foremost, the catalyst-to-photosensitizer ratio is a determining factor for the photocatalytic hydrogen evolution with lower catalyst loadings leading to the highest TON per catalyst. This is particularly attractive if the goal is to minimize catalyst input and maximize the activity of the single catalytic sites. However, an alternate viewpoint emerges when we consider hydrogen production per nanorod, with photocatalytic system IV outperforming the others, emphasizing the significance of catalyst loading on a per-nanorod basis. Low TONs per catalyst might still be overcompensated in systems with high catalyst loadings, in terms of absolute hydrogen production. This shows that for optimal performance of the systems, individual optimal loadings for each of the investigated catalysts need to be determined and precise control of catalyst loading needs to be achieved in future functionalization schemes. These optimizations would lead to an optimum between the amount of catalyst loading and the supply of excited electrons to the catalytic center. However, when considering the overall water splitting process, these optimizations would also have to be adapted to the respective oxidation half-reaction.

## Conclusions

In conclusion, this study presents a comprehensive investigation of a novel photocatalytic system comprising CdSe@CdS nanorods as photosensitizers coated with polydopamine (PDA), a photoprotective matrix, and various cobalt-based catalysts. Our approach opens the possibility of introducing molecular catalysts, which are not water-soluble, to an aqueous solution by attaching them to the PDA-coated CdSe@CdS nanorods. The choice of catalyst, its loading, and the consequent impact on both hydrogen production and electron supply have been elucidated. These findings offer a foundation for optimizing photocatalytic systems and contribute to a broader understanding of catalyst-nanorod interactions in renewable energy applications. We find that in the investigated systems, the overall performance strongly depends on catalyst loading, affecting the individual activity of a single catalytic site. Furthermore, surface coverage contributed more to the catalyst activity than their intrinsic reactivity when it was not bound to a nanoparticle surface. While the individual catalyst activity depends on the number of surface-attached catalysts, the overall hydrogen production per nanorod can be increased by simply loading more catalysts to the surface. The limit at which increasing catalyst loading does not further increase hydrogen production is probably characteristic of each system and must be determined individually. In contrast to the metal nanoparticle-based cocatalyst system, where lower catalyst loadings appear more efficient,^43^ our results indicate a balance between molecular catalyst loading and the supply of excited electrons to the catalytic center. Thus, precise control of the catalyst loading should be achieved in future functionalization approaches.

## Supporting Information Available

Materials and synthesis, instruments and techniques, STEM-ADF images, TEM images, EELS mapping of the NR and PDA elements for the cNR, XPS spectra, TXRF results, molar ratio of Co to Cd for the initial photocatalytic system and after 1 week of storing determined by TXRF, and stability of hydrogen production efficiency after 3 weeks of storage (PDF)

## Abbreviations

NR: CdSe@CdS nanorod
PDA: polydopamine
cNR: polydopamine-coated CdSe@CdS nanorod
cNR-PEG: polydopamine-coated CdSe@CdS nanorods functionalized with polyethylene glycol
cNR-PEG-Cat: polydopamine-coated CdSe@CdS nanorods functionalized with polyethylene glycol and with one associated cobalt-based catalyst
XPS: X-ray photoelectron spectroscopy
TXRF: total reflection X-ray fluorescence spectrometry
TON: turnover number
HER: hydrogen evolution reaction
OER: oxygen evolution reaction
NHE: normal hydrogen electrode
NAD^+^: β-nicotinamide adenine dinucleotide hydride, oxidized form
NADH: β-nicotinamide adenine dinucleotide hydride, reduced form
SEM: scanning electron microscopy

## References

[ref1] Edwards P. P.; Kuznetsov V. L.; David W. I. F. Hydrogen Energy. Phil Trans R Soc. A 2007, 365 (1853), 1043–1056. 10.1098/rsta.2006.1965. 17272235

[ref2] Abe J. O.; Popoola A. P. I.; Ajenifuja E.; Popoola O. M. Hydrogen Energy, Economy and Storage: Review and Recommendation. Int. J. Hydrogen Energy 2019, 44 (29), 15072–15086. 10.1016/j.ijhydene.2019.04.068.

[ref3] Hren R.; Vujanović A.; Van Fan Y.; Klemeš J. J.; Krajnc D.; Čuček L. Hydrogen Production, Storage and Transport for Renewable Energy and Chemicals: An Environmental Footprint Assessment. Renew. Sustain. Energy Rev. 2023, 173, 113113 10.1016/j.rser.2022.113113.

[ref4] Yang H.; Hou H.; Yang M.; Zhu Z.; Fu H.; Zhang D.; Luo Y.; Yang W. Engineering the S-Scheme Heterojunction between NiO Microrods and MgAl-LDH Nanoplates for Efficient and Selective Photoreduction of CO2 to CH4. Chem. Eng. J. 2023, 474, 145813 10.1016/j.cej.2023.145813.

[ref5] Yang H.; Gao J.; Yang M.; Hou H.; Gao F.; Luo Y.; Yang W. One-Pot MOFs-Encapsulation Derived In-Doped ZnO@In2O3 Hybrid Photocatalyst for Enhanced Visible-Light-Driven Photocatalytic Hydrogen Evolution. Adv. Sustain. Syst. 2023, 7 (4), 2200443 10.1002/adsu.202200443.

[ref6] Zhu S.; Wang D. Photocatalysis: Basic Principles, Diverse Forms of Implementations and Emerging Scientific Opportunities. Adv. Energy Mater. 2017, 7 (23), 1700841 10.1002/aenm.201700841.

[ref7] Kim J. H.; Hansora D.; Sharma P.; Jang J. W.; Lee J. S. Toward Practical Solar Hydrogen Production-an Artificial Photosynthetic Leaf-to-Farm Challenge. Chem. Soc. Rev. 2019, 48 (7), 1908–1971. 10.1039/C8CS00699G. 30855624

[ref8] Christoforidis K. C.; Fornasiero P. Photocatalytic Hydrogen Production: A Rift into the Future Energy Supply. ChemCatChem 2017, 9 (9), 1523–1544. 10.1002/cctc.201601659.

[ref9] Teets T. S.; Nocera D. G. Photocatalytic Hydrogen Production. ChemComm 2011, 47 (33), 9268–9274. 10.1039/c1cc12390d. 21647489

[ref10] Zhang K.; Guo L. Metal Sulphide Semiconductors for Photocatalytic Hydrogen Production. Catal. Sci. Technol. 2013, 3 (7), 1672–1690. 10.1039/c3cy00018d.

[ref11] El-Sayed M. A. Small Is Different: Shape-Size-and Composition-Dependent Properties of Some Colloidal Semiconductor Nanocrystals. Acc. Chem. Res. 2004, 37 (5), 326–333. 10.1021/ar020204f. 15147173

[ref12] She C.; Demortière A.; Shevchenko E. V.; Pelton M. Using Shape to Control Photoluminescence from CdSe/CdS Core/Shell Nanorods. J. Phys. Chem. Lett. 2011, 2 (12), 1469–1475. 10.1021/jz200510f.

[ref13] Micheel M.; Liu B.; Wächtler M. Influence of Surface Ligands on Charge-Carrier Trapping and Relaxation in Water-Soluble Cdse@cds Nanorods. Catalysts 2020, 10 (10), 1143 10.3390/catal10101143.

[ref14] Amirav L.; Alivisatos A. P. Photocatalytic Hydrogen Production with Tunable Nanorod Heterostructures. J. Phys. Chem. Lett. 2010, 1 (7), 1051–1054. 10.1021/jz100075c.

[ref15] Wächtler M.; Kalisman P.; Amirav L. Charge-Transfer Dynamics in Nanorod Photocatalysts with Bimetallic Metal Tips. J. Phys. Chem. C 2016, 120 (42), 24491–24497. 10.1021/acs.jpcc.6b09265.

[ref16] Chica B.; Wu C. H.; Liu Y.; Adams M. W. W.; Lian T.; Dyer R. B. Balancing Electron Transfer Rate and Driving Force for Efficient Photocatalytic Hydrogen Production in CdSe/CdS Nanorod-[NiFe] Hydrogenase Assemblies. Energy Environ. Sci. 2017, 10 (10), 2245–2255. 10.1039/C7EE01738C.

[ref17] Wolff C. M.; Frischmann P. D.; Schulze M.; Bohn B. J.; Wein R.; Livadas P.; Carlson M. T.; Jäckel F.; Feldmann J.; Würthner F.; Stolarczyk J. K. All-in-One Visible-Light-Driven Water Splitting by Combining Nanoparticulate and Molecular Co-Catalysts on CdS Nanorods. Nat. Energy 2018, 3 (10), 862–869. 10.1038/s41560-018-0229-6.

[ref18] Jasieniak J.; Mulvaney P. From Cd-Rich to Se-Rich - The Manipulation of CdSe Nanocrystal Surface Stoichiometry. J. Am. Chem. Soc. 2007, 129 (10), 2841–2848. 10.1021/ja066205a. 17309253

[ref19] Manner V. W.; Koposov A. Y.; Szymanski P.; Klimov V. I.; Sykora M. Role of Solvent-Oxygen Ion Pairs in Photooxidation of CdSe Nanocrystal Quantum Dots. ACS Nano 2012, 6 (3), 2371–2377. 10.1021/nn2046289. 22381115

[ref20] Liebscher J.; Mrówczyński R.; Scheidt H. A.; Filip C.; Hădade N. D.; Turcu R.; Bende A.; Beck S. Structure of Polydopamine: A Never-Ending Story?. Langmuir 2013, 29 (33), 10539–10548. 10.1021/la4020288. 23875692

[ref21] Aguilar-Ferrer D.; Szewczyk J.; Coy E. Recent Developments in Polydopamine-Based Photocatalytic Nanocomposites for Energy Production: Physico-Chemical Properties and Perspectives. Catal. Today 2022, 397–399, 316–349. 10.1016/j.cattod.2021.08.016.

[ref22] Lee H.; Dellatore S. M.; Miller W. M.; Messersmith P. B. Mussel-Inspired Surface Chemistry for Multifunctional Coatings. Science (1979) 2007, 318 (5849), 426–430. 10.1126/science.1147241. PMC2601629 17947576

[ref23] Kim Y.; Coy E.; Kim H.; Mrówczyński R.; Torruella P.; Jeong D. W.; Choi K. S.; Jang J. H.; Song M. Y.; Jang D. J.; Peiro F.; Jurga S.; Kim H. J. Efficient Photocatalytic Production of Hydrogen by Exploiting the Polydopamine-Semiconductor Interface. Appl. Catal., B 2021, 280, 119423 10.1016/j.apcatb.2020.119423.

[ref24] Wang M.; Cui Z.; Yang M.; Lin L.; Chen X.; Wang M.; Han J. Core/Shell Structured CdS/Polydopamine/TiO 2 Ternary Hybrids as Highly Active Visible-Light Photocatalysis. J. Colloid Interface Sci. 2019, 544, 1–7. 10.1016/j.jcis.2019.02.080. 30818155

[ref25] Ruan M.; Guo D.; Jia Q. A Uniformly Decorated and Photostable Polydopamine-Organic Semiconductor to Boost the Photoelectrochemical Water Splitting Performance of CdS Photoanodes. Dalton Trans. 2021, 50 (5), 1913–1922. 10.1039/D0DT04056H. 33475654

[ref26] Kim J. H.; Lee M.; Park C. B. Polydopamine as a Biomimetic Electron Gate for Artificial Photosynthesis. Angew. Chem. Inter. Ed. 2014, 53 (25), 6364–6368. 10.1002/anie.201402608. 24692071

[ref27] Lee H.; Rho J.; Messersmith P. B. Facile Conjugation of Biomolecu Les onto Surfaces via Mussel Adhesive Protein Inspired Coatings. Adv. Mater. 2009, 21 (4), 431–434. 10.1002/adma.200801222. 19802352 PMC2755254

[ref28] Xu L. Q.; Yang W. J.; Neoh K. G.; Kang E. T.; Fu G. D. Dopamine-Induced Reduction and Functionalization of Graphene Oxide Nanosheets. Macromol. 2010, 43 (20), 8336–8339. 10.1021/ma101526k.

[ref29] Boecker M.; Micheel M.; Mengele A. K.; Neumann C.; Herberger T.; Marchesi D’Alvise T.; Liu B.; Undisz A.; Rau S.; Turchanin A.; Synatschke C. V.; Wächtler M.; Weil T. Rhodium-Complex-Functionalized and Polydopamine-Coated CdSe@CdS Nanorods for Photocatalytic NAD+ Reduction. ACS Appl. Nano Mater. 2021, 4 (12), 12913–12919. 10.1021/acsanm.1c02994. 34977477 PMC8713362

[ref30] Wang W.; Li T.; Komarneni S.; Lu X.; Liu B. Recent Advances in Co-Based Co-Catalysts for Efficient Photocatalytic Hydrogen Generation. J. Colloid Interface Sci. 2022, 608, 1553–1575. 10.1016/j.jcis.2021.10.051. 34742073

[ref31] Dolui D.; Khandelwal S.; Majumder P.; Dutta A. The Odyssey of Cobaloximes for Catalytic H _2_ Production and Their Recent Revival with Enzyme-Inspired Design. ChemComm 2020, 56 (59), 8166–8181. 10.1039/D0CC03103H. 32555820

[ref32] Cartwright K. C.; Davies A. M.; Tunge J. A. Cobaloxime-Catalyzed Hydrogen Evolution in Photoredox-Facilitated Small-Molecule Functionalization. Eur. J. Org Chem. 2020, 2020 (10), 1245–1258. 10.1002/ejoc.201901170.

[ref33] Cropek D. M.; Metz A.; Müller A. M.; Gray H. B.; Horne T.; Horton D. C.; Poluektov O.; Tiede D. M.; Weber R. T.; Jarrett W. L.; Phillips J. D.; Holder A. A. A Novel Ruthenium(Ii)-Cobaloxime Supramolecular Complex for Photocatalytic H2 Evolution: Synthesis, Characterisation and Mechanistic Studies. Dalton Trans. 2012, 41 (42), 13060 10.1039/c2dt30309d. 23001132 PMC3482109

[ref34] Xu Y.; Chen R.; Li Z.; Li A.; Han H.; Li C. Influence of the Electrostatic Interaction between a Molecular Catalyst and Semiconductor on Photocatalytic Hydrogen Evolution Activity in Cobaloxime/CdS Hybrid Systems. ACS Appl. Mater. Interfaces 2017, 9 (27), 23230–23237. 10.1021/acsami.7b06154. 28631477

[ref35] Schleusener A.; Micheel M.; Benndorf S.; Rettenmayr M.; Weigand W.; Wächtler M. Ultrafast Electron Transfer from CdSe Quantum Dots to an [FeFe]-Hydrogenase Mimic. J. Phys. Chem. Lett. 2021, 12 (18), 4385–4391. 10.1021/acs.jpclett.1c01028. 33939438

[ref36] Benndorf S.; Schleusener A.; Müller R.; Micheel M.; Baruah R.; Dellith J.; Undisz A.; Neumann C.; Turchanin A.; Leopold K.; Weigand W.; Wächtler M. Covalent Functionalization of CdSe Quantum Dot Films with Molecular [FeFe] Hydrogenase Mimics for Light-Driven Hydrogen Evolution. ACS Appl. Mater. Interfaces 2023, 15 (15), 18889–18897. 10.1021/acsami.3c00184. 37014708 PMC10120591

[ref37] Oswald E.; Gaus A. L.; Kund J.; Küllmer M.; Romer J.; Weizenegger S.; Ullrich T.; Mengele A. K.; Petermann L.; Leiter R.; Unwin P. R.; Kaiser U.; Rau S.; Kahnt A.; Turchanin A.; von Delius M.; Kranz C. Cobaloxime Complex Salts: Synthesis, Patterning on Carbon Nanomembranes and Heterogeneous Hydrogen Evolution Studies. Chem.—Eur. J. 2021, 27 (68), 16896–16903. 10.1002/chem.202102778. 34713512 PMC9299159

[ref38] Richard-Lacroix M.; Küllmer M.; Gaus A. L.; Neumann C.; Tontsch C.; von Delius M.; Deckert V.; Turchanin A. Synthesis and Nanoscale Characterization of Hierarchically Assembled Molecular Nanosheets. Adv. Mater. Interfaces 2022, 9 (14), 2102389 10.1002/admi.202102389.

[ref39] Kund J.; Romer J.; Oswald E.; Gaus A. L.; Küllmer M.; Turchanin A.; von Delius M.; Kranz C. Pd-Modified De-Alloyed Au-Ni-Microelectrodes for In Situ and Operando Mapping of Hydrogen Evolution. ChemElectroChem. 2022, 9 (6), e202200071 10.1002/celc.202200071.

[ref40] Fihri A.; Artero V.; Razavet M.; Baffert C.; Leibl W.; Fontecave M. Cobaloxime-Based Photocatalytic Devices for Hydrogen Production. Angew. Chem. Inter. Ed. 2008, 47 (3), 564–567. 10.1002/anie.200702953. 18095368

[ref41] Dempsey J. L.; Brunschwig B. S.; Winkler J. R.; Gray H. B. Hydrogen Evolution Catalyzed by Cobaloximes. Acc. Chem. Res. 2009, 42 (12), 1995–2004. 10.1021/ar900253e. 19928840

[ref42] Losse S.; Vos J. G.; Rau S. Catalytic Hydrogen Production at Cobalt Centres. Coord. Chem. Rev. 2010, 254 (21–22), 2492–2504. 10.1016/j.ccr.2010.06.004.

[ref43] Nakibli Y.; Kalisman P.; Amirav L. Less Is More: The Case of Metal Cocatalysts. J. Phys. Chem. Lett. 2015, 6 (12), 2265–2268. 10.1021/acs.jpclett.5b00872. 26266602

[ref44] Simon T.; Carlson M. T.; Stolarczyk J. K.; Feldmann J. Electron Transfer Rate vs Recombination Losses in Photocatalytic H2 Generation on Pt-Decorated CdS Nanorods. ACS Energy Lett. 2016, 1 (6), 1137–1142. 10.1021/acsenergylett.6b00468.

