# Supplementary material for: Screening Cobalt-based Catalysts on Multicomponent CdSe@CdS Nanorods for Photocatalytic Hydrogen Evolution in Aqueous Media
Source: ACS Appl Nano Mater. 2024 Jun 18;7(12):14146–53. doi: 10.1021/acsanm.4c01645 (PMC11217917; doi:10.1021/acsanm.4c01645)
Supplement: Supplementary file 1 — an4c01645_si_001.pdf [file an4c01645_si_001.pdf]

Supporting information for

Screening Cobalt-Based Catalysts on Multicomponent CdSe@CdS Nanorods for Photocatalytic Hydrogen Evolution in Aqueous Media

Marcel Boecker[a], Sarah Lander[b], Riccarda Müller[c], Anna-Laurine Gaus[d], Christof Neumann[e], Julia Moser[a], Mathias Micheel[b], Andrey Turchanin[e,f], Max von Delius[d], Christopher V. Synatschke[a], Kerstin Leopold[c], Maria Wächtler*[,b], Tanja Weil*[,a]

[a] Department for Synthesis of Macromolecules, Max Planck Institute for Polymer Research, 55128 Mainz, Germany

[b] Department of Chemistry and State Research Center OPTIMAS, RPTU Kaiserslautern-Landau, 67663 Kaiserslautern, Germany

[c] Institute of Analytical and Bioanalytical Chemistry, University Ulm, 89081 Ulm, Germany

[d] Institute of Organic Chemistry I, University Ulm, 89081 Ulm, Germany

[e] Institute of Physical Chemistry, Friedrich Schiller University Jena, 07743 Jena, Germany

[f] Abbe Center of Photonics (ACP), 07745 Jena, Germany

Corresponding Author

Tanja Weil – Department for Synthesis of Macromolecules, Max Planck Institute for polymer research, 55128 Mainz, Germany; Email: weil@mpip-mainz.mpg.de

Maria Wächtler – Department of Chemistry and State Research Center OPTIMAS, RPTU Kaiserslautern-Landau, 67663 Kaiserslautern, Germany; Email: maria.waechtler@chem.rptu.de

Table of content

1. Materials and Synthesis 1

2. Instruments and techniques 3

3. Supporting figures 10

4. References 25

1. Materials and Synthesis

Materials. All chemicals and solvents were purchased from Sigma Aldrich (Merck KGaA, Darmstadt, Germany) except for 1-ethyl-3-(3-dimethylaminopropyl)carbodiimide (EDC) which was purchased from G-Biosciences (St. Louis, USA) and octadecylphosphonic acid (ODPA) which was purchased from Carl Roth GmbH + Co. KG (Karlsruhe, Germany). All reactions were performed under air if not otherwise stated.

Buffer solutions. Buffer solutions were prepared according to our previous publication.[1] Tris-HCl buffer (0.1 M, pH = 8.5 or 7.5) was prepared by dissolving Tris-(hydroxymethyl) aminomethane (121.14 g/mol, 0.005 mol) in 50 mL MilliQ water, and the pH was adjusted to 8.5 with 1 M HCl. Phosphate buffer (0.1 M, pH = 7) was prepared by dissolving sodium phosphate dibasic (anhydrous, 141.96 g/mol, 0.0482 mol) and sodium phosphate monobasic (anhydrous, 119.98 g/mol, 0.0018 mol) in 500 mL MilliQ water, and the pH was adjusted with 1 M HCl or 1 M NaOH if necessary. The pH was checked and adjusted for all buffer solutions using a pH meter (Mettler Toledo MP220 pH meter).

Synthesis of CdSe seeds. The CdSe seed synthesis was adapted from a known procedure in literature.[2] 3.00 g trioctylphosphine oxide (TOPO), 0.28 g octadecylphosphonic acid (ODPA), and 0.06 g CdO were placed in a 25-mL-three-neck-flask. The reaction was performed under an inert atmosphere and constant stirring. The flask was heated to 80 °C to melt the chemicals and evacuated to remove water in the chemicals. Once no more gas emerged from the solution, the flask was heated to 120 °C and kept evacuated for 30 min. After that, the flask was purged with N_2. The flask was then heated to 320 °C, upon which the solution turned colorless due to the complexation of Cd-ODPA, and then cooled down to 120 °C. Then, a vacuum was applied to remove water, a side product of the complexation of Cd-ODPA; after gas formation in the reaction mixture stopped (at least 2 h), then, the flask was purged with N_2 and heated to 340 °C. Next, 0.058 g Se dissolved in 0.36 g trioctyl phosphine (TOP) were injected. After the injection, the heating was immediately stopped. After the temperature of the mixture was cooled to 90 °C, 5 mL of toluene was injected into the mixture. The seeds were cleaned five times by centrifugation with 10 mL toluene in 10 mL methanol and then dissolved in toluene. The diameter of the CdSe seeds was determined from the energetic position of the absorption peak with the lowest energy[3] to be 2 nm.

Synthesis of the CdSe@CdS nanorods (NR). The CdSe@CdS nanorods were synthesized according to a reported protocol in literature.[4] 3.35 g trioctylphosphine oxide (TOPO), 1.08 g octadecylphosphonic acid (ODPA), 0.207 g CdO, and 0.06 g *n*-propylphosphonic acid (PPA) were placed in a 25-mL-three-neck-flask. The reaction was performed under an inert atmosphere and constant stirring. The flask was heated up to 80 °C until the reaction mixture melted and evacuated to remove residual water from the mixture. Once gas formation stopped, the flask was heated to 120 °C and kept evacuated for 30 min. After that, the flask was flooded with N_2. The flask was then heated to 320 °C, upon which the solution turned colorless due to the complexation of Cd-ODPA, and then cooled down to 120 °C. Next, a vacuum was applied until gas formation in the reaction mixture stopped (at least 2 h) to remove water, which is a side product of Cd-ODPA complexation. Then, the flask was flooded with N_2 again and heated up to 340 °C. Next, 1.5 g TOPO and 0.05 g sulfur dissolved in 0.60 g TOP were injected. After 20 s, 2 mg of CdSe seeds (diameter = 2.0 nm) dissolved in 0.5 g TOPO were injected. The reaction was allowed to stir for 10 min until the color of the solution turned from red to orange. 5 mL toluene was injected into the mixture once the temperature dropped below the flashing point of toluene to stop solidification of the mixture. After precipitation with 10 mL methanol, rods were cleaned five times by centrifugation with 6 mL

n-hexane, 2 mL nonanoic acid, and 2 mL octylamine in 10 mL methanol. Next, the rods were separated according to size by centrifugation at 4200 rpm for 30 min with 10 mL toluene and 8 mL isopropanol to obtain rods with lengths of circa 40 nm. This was repeated two times in total. The NR were then dispersed in toluene for further investigation.

Ligand exchange with 11-mercaptoundecanoic acid (MUA). The ligand exchange with MUA was performed according to a published protocol.[5] 250 mg MUA were dissolved in 25 mL methanol and tetramethylammonium hydroxide pentahydrate was added until the solution reached pH 11 (circa 200 mg). 20 mg NR (dried from its toluene solution under vacuum) were added into this mixture and stirred for 2 h. When the NR were fully dispersed, approximately 35 mL of toluene was added as non-solvent until NR precipitated. The mixture was then centrifuged at 6000 rpm for 20 min, the supernatant discarded, and the precipitate was redispersed in degassed water.

The synthesis of the cobalt catalysts was performed according to the previously published method.[6]

PDA-coated nanorods (cNR). Coating of the CdSe@CdS nanorods with PDA was performed according to our previous publication.[1] 50 µL of the nanorod solution (3 mg/mL) were diluted with 400 µL of Tris-HCl buffer (0.1 M, pH = 8.5) in a 1.5 mL Eppendorf tube and 50 µL of dopamine solution (1 mg/mL in MilliQ water) were added. The Eppendorf tube was wrapped in aluminum foil to prevent light exposure. Then, the reaction solution was vortexed for 24 h at 1000 rpm at room temperature. For purification, the reaction solution was filtered through a centrifuge filter at 4000 g for 5 min to remove small molecule residuals like free dopamine. The coated nanorods remained in the filter and were redispersed in MilliQ water. The filtration was repeated twice. As centrifuge filters Millipore centrifugal filter units (Amicon Ultra – 0.5 mL, Ultracel – 100k) with a cut-off of 100 kDa and a centrifuge (Heraeus Fresco 2, Thermo Fisher Scientific) were used. For further functionalization, the remaining PDA-coated nanorods were redispersed in phosphate buffer (0.1 M, pH = 8.5) while they were redispersed in MilliQ for storage.

Functionalization of cNR with PEG. 1 mg of O-[2-(3-Mercaptopropionylamino)-ethyl]-O′-methyl-polyethylenglycol 5,000 was added to 0.5 mL of the cNR in phosphate buffer (0.1 M, pH = 8.5) in a 1.5 mL Eppendorf tube. The Eppendorf tube was wrapped in Aluminium foil to prevent light exposure and the solution was vortexed for 24 h at 1000 rpm. Purification was done by spin-filtration of the reaction solution 3 times through a centrifuge filter at 4000 g for 5 min, the remaining PEGylated cNRs were redispersed in water. The same centrifuge filters as for the previous purification were used. After the last purification cycle, the remaining cNR-PEG were redispersed in phosphate buffer (0.1 M, pH = 7).

Functionalization of the cNR-PEG with isonicotinic acid. The functionalization of the cNR-PEG with isonicotinic acid was adapted from a previous publication.[1] Therefore, isonicotinic acid (0.3 mg, 2.4 µmol), 1-ethyl-3-(3-dimethylaminopropyl)carbodiimide (EDC, 1.2 eq.) and *N*-hydroxysuccinimide (NHS, 2 eq.) were added to 0.5 mL of the cNR-PEG in phosphate buffer (0.1 M, pH = 7) in an Eppendorf tube, which was wrapped with aluminum foil to prevent light exposure. The reaction solution was vortexed for 24 h at 1000 rpm. Purification was done by 3 times filtering through a centrifuge filter at 4000 g for 5 min, the remaining functionalized nanorods were redispersed in MilliQ water. After the last spin-filtration, the remaining functionalized nanorods were redispersed in 0.4 mL phosphate buffer (0.1 M, pH = 7).

Attachment of catalyst. To ensure that the same coating for NR was used for all five catalysts, a large batch was split into 5 reaction vessels. The respective catalysts were associated by adding

0.1 mL of the respective catalysts (1 µmol/mL) in acetonitrile to 0.4 mL of the functionalized cNR-PEG in phosphate buffer (0.1 M, pH = 7) in an Eppendorf tube. The Eppendorf tube was wrapped in aluminum foil to prevent light exposure and vortexed for 24 h at 1000 rpm. For purification, the reaction solution was 3 times filtered through a centrifugal filter at 4000 g for 5 min. The remaining cNR-PEG-cat were redispersed in MilliQ water.

As a control experiment, the different catalysts (same association solution as for the photocatalytic samples) were also mixed with the cNR-PEG samples (0.4 mL). The samples were placed on vortexer for 24 h at 1000 rpm. The purification was done analog to the photocatalytic samples.

2. Instruments and techniques

Energy-dispersive X-ray spectroscopy (EDX): The EDX spectrum was recorded on a Hitachi SU8000 scanning electron microscope (Hitachi High-Technologies Europe GmbH, Krefeld) with a Bruker Quantax EDX device. For the measurements, a primary energy of 8 keV, a tilt angle of 0°, a take-off angle of 30° and as detector a Bruker XFlash 5010 (fifth generation, 10 mm^2 detector area) was used. The data was analyzed using the Bruker Quantax Esprit 2.3 software.

X-ray photoelectron spectroscopy (XPS): For XPS analysis, each sample was drop-cast on a Si wafer for the functionalization steps or Au substrate for the catalytic systems. The measurements were performed using a K-Alpha X-ray Photoelectron Spectrometer System (Thermo Fisher Scientific) with a monochromatic X-ray source (Al K_α) with a spot diameter of 400 µm and an electron detector with 0.5 eV energy resolution. The spectra were calibrated using the Au $4f_{7/2}$ peak (84.0 eV) and the Si 2p peak (SiO_2, 103.6 eV), respectively, and fitted using Voigt functions after background subtraction. For the Co 2p spectra, only the main component is shown due to the low signal-to-noise ratio.

Hydrogen measurements: To quantify the hydrogen evolution, 20µl of the respective catalyst-functionalized cNR-PEG solutions were added to 2mL degassed ascorbic acid buffer solution (0.1 M, pH = 5) in gas-tight glass vials. These samples were irradiated with a 455nm LED (ThorLabs GmbH) with 3 mW/cm^2 for 6 h at RT. Uniform light distribution was ensured by the reactor design. 100 µL of the headspace were used for hydrogen quantification via GC-TCD.

Total reflection X-ray spectrometry (TXRF): Quantification of Co on catalyst functionalized CdSe@CdS rods was performed using TXRF high-efficiency module S2 Picofox (Bruker Nano GmbH, Berlin, Germany) equipped with Mo X-ray tube. Excitation of the sample was carried out using a voltage of 50 kV and a current of 450 µA. Measurement live time was set to 1000 s. Evaluation of the obtained spectra was achieved using Spectra PicoFox (7.2.5.0, Bruker Nano GmbH) software and for deconvolution the profile bayes normal fit was selected.

Samples were prepared as follows: A) Initial, unfiltered suspensions of functionalized CdSe@CdS rods in water were investigated directly after preparation and after 7 days of storage. In both cases, suspensions were homogenized using a Vortex mixer (60 s; 2500 rpm) and then 50 µL (Co+Co-, commercial, TBA, CoBarF) or 25 µL (CoBPh2) were diluted in 500 µL ultra pure water (UPW).

B) Suspensions of functionalized CdSe@CdS rods in water (freshly prepared and after 7 days of storage) were purified using spin filtration. To study the stability of the Co catalyst on the rods filtrate and purified samples were investigated for their Co content. For this purpose, 350 µL of the unfiltered initial suspension were spin filtered (Amicon Ultra – 0.5 mL 100 K, Merck KGaA, Darmstadt, Germany)

for 5 min at 4000 g and 20 °C. The purified rods were re-suspended in 300 µL UPW and transferred to a 2 mL cup. Filtrate and purified rods were diluted appropriately in UPW.

For Co quantification in these samples, 10 µL of Ti standard solution (100 mg L^{-1} in 2 wt.-% HNO_3, Honeywell Fluka, Charlotte, North Carolina, USA) as an internal standard were added. After vigorous mixing, 10 µL of each sample were applied to a quartz glass sample carrier which was then placed onto a heating plate at 60 °C for evaporation of solvent until complete dryness. For each sample, three sample carriers were prepared (n=3).

Calculation of the number of nanorods based on measured Cd concentration:

The dimensions of the CdSe@CdS rods were determined by the analysis of 640 individual particles imaged by SEM and probed in length and width using FIJI (ImageJ v. 1.53c.). The resulting size histograms (Figure S8) yielded an average length of 43.8 ± 5.8 nm and width of 4.8 ± 0.4 nm. The volumes of CdS and CdSe unit cells in a wurtzite crystal structures are 102 Å and 112 Å respectively.[7] The volume of the components of the dot-in-rod divided by their respective unit cell volume and multiplied by the number of atoms per unit cell, thus resulting in an estimate of the number of Cd atoms per nanorod. This was determined to be $2x10^{-20}$ mol of Cd per nanorod. The measured Cd concentrations can then be directly translated into the number of nanorods in solution.

Calculation of catalyst per NR $\left(\frac{\#Co}{NR}\right)$*:* To calculate the number of catalysts per NR, the measured molar amount of Co ($n(Co)$) by TXRF was multiplied with the Avogadro constant (N_A) and divided by the calculated amount of NR (#NR, for calculation see above).

$$\frac{\#Co}{NR} = \frac{n(Co) \cdot N_A}{\#NR} \tag{1}$$

Calculation of TON per Co center $\left(TON(\text{per Co})\right)$*:* To calculate the TON per Co center, the measured molar H_2 amount $\left(n(H_2)\right)$by GC was divided by the determined molar amount of Co ($n(Co)$) per sample by TXRF. Since three photocatalytic runs were performed, the mean value of the 3 calculated TONs was calculated after and reported.

$$TON(\text{per Co}) = \frac{n(H_2)}{n(Co)} \tag{2}$$

Calculation of H_2 per NR: To calculate the number of hydrogen molecules per NR $\left(\frac{\#H_2}{NR}\right)$ the previously calculated $TON(\text{per Co})$ was multiplied with the calculated molar amount of Co per NR $\left(\frac{n(\text{Co per NR})}{\#NR}\right)$ and the Avogadro constant (N_A) to give the amount of H_2 per nanorod.

$$\frac{\#H_2}{NR} = TON(\text{per Co}) \cdot \frac{n(\text{Co per NR})}{\#NR} \cdot N_A \tag{3}$$

Table S 1: Quantitative analysis of the high-resolution XP spectra presented in Figure S4-S12 including the peak assignment, their binding energies, full width at half maximum (FWHM) values and areas obtained from the spectra deconvolution. The peak fitting of the S and Co doublets was performed using fixed intensity ratios due to the spin-orbit coupling of the p photoelectrons.

Peak assignment	Binding energy, eV	FWHM, eV	Area, %
C 1s			
cNR			
C-C/C-H_x	284.6	1.3	72.9
C-O/C-N	286.1	1.3	19.6
C=O/COOH	287.9	2.0	7.5
cNR with isonicotinic acid functionalized			
C-C/C-H_x	284.6	1.4	48.0
C-O/C-N	285.8	2.0	37.9
C=O/COOH	288.2	2.0	14.1
cNR-PEG			
C-C/C-H_x	284.6	1.3	44.9
C-O/C-N	286.1	2.0	42.0
C=O/COOH	288.2	2.0	13.1
cNR-PEG and isonicotinic acid			
C-C/C-H_x	284.6	1.4	43.5
C-O/C-N	286.0	2.0	43.3
C=O/COOH	288.2	2.0	13.2
cNR-PEG-$[Co(dmgH)_2(py)Cl]$			
C-C/C-H_x	284.6	1.3	67.7
C-O/C-N	286.1	1.3	23.2
C=O/COOH	287.7	2.2	9.1
NR-PEG-$[Co]^+$ $[Co]^-$			
C-C/C-H_x	284.6	1.3	68.2
C-O/C-N	286.1	1.3	23.7
C=O/COOH	287.8	1.9	8.1
cNR-PEG-TBA^+ $[Co(BPh_2)_2]^-$			
C-C/C-H_x	284.6	1.7	64.4
C-O/C-N	286.2	1.8	24.7
C=O/COOH	287.5	3.5	10.9
cNR-PEG-$[Co]^+$ $[Co(BPh_2)_2]^-$			
C-C/C-H_x	284.6	1.5	36.8
C-O/C-N	286.2	1.3	54.0
C=O/COOH	287.5	2.0	9.2
cNR-PEG-$[Co]^+$ $BArF^-$			
C-C/C-H_x	284.6	1.3	64.4
C-O/C-N	286.1	1.3	27.9
C=O/COOH	287.7	2.0	7.7

Peak assignment	Binding energy, eV	FWHM, eV	Area, %
N 1s			
cNR			
=NR	398.6	1.2	32.3
RNH_2	399.4	1.1	67.7
cNR with isonicotinic acid functionalized			
=NR	399.5	1.4	34.7
RNH_2	400.4	1.4	65.3
cNR-PEG			
=NR	399.6	1.4	30.3
RNH_2	400.4	1.4	69.7
cNR-PEG and isonicotinic acid			
=NR	399.7	1.4	49.9
RNH_2	400.4	1.4	50.1
cNR-PEG-[Co(dmgH)2(py)Cl]			
=NR	398.7	1.2	60.1
RNH_2	399.7	1.2	39.9
cNR-PEG-[Co]+ [Co]-			
=NR	398.7	1.2	35.3
RNH2	399.6	1.2	64.7
cNR-PEG-TBA+ [Co(BPh2)2]-			
=NR	399.0	1.4	41.6
RNH2	400.1	1.2	58.4
cNR-PEG-[Co]+ [Co(BPh2)2]-			
=NR	398.3	1.2	27.4
RNH2	399.5	1.2	72.6
cNR-[Co]+ BArF-			
=NR	398.4	1.1	32.9
RNH2	399.6	1.2	67.1

Peak assignment	Binding energy, eV	FWHM, eV	Area, %
O 1s			
cNR			
O=C	531.0	1.6	34.7
O-C/SiO2	532.5	1.7	61.9
-OH	534.4	2.0	3.4
cNR with isonicotinic acid functionalized			
O=C	531.5	1.8	49.2
O-C/SiO2	532.5	1.9	33.9
-OH	533.6	2.0	16.9
cNR-PEG			
O=C	531.3	1.7	34.6
O-C/SiO2	532.7	2.0	54.4
-OH	533.9	2.0	11.0
cNR-PEG and isonicotinic acid			
O=C	531.4	1.8	36.3
O-C/SiO2	532.6	2.0	55.7
-OH	534.2	2.0	8.0
cNR-PEG-[Co(dmgH)2(py)Cl]			
O=C	530.6	1.6	57.1
O-C/SiO2	532.4	1.7	38.4
-OH	533.0	1.9	4.5
cNR-PEG-[Co]+ [Co]-			
O=C	530.8	1.7	53.7
O-C/SiO2	532.5	1.6	41.9
-OH	533.5	2.0	4.4
cNR-PEG-TBA+ [Co(BPh2)2]-			
O=C	530.9	1.8	41.2
O-C/SiO2	532.5	2.0	51.3
-OH	533.3	1.7	7.5
cNR-PEG-[Co]+ [Co(BPh2)2]-			
O=C	530.6	1.7	35.1
O-C/SiO2	532.5	1.5	56.0
-OH	533.0	2.0	8.9
cNR-[Co]+ BArF-			
O=C	530.7	1.6	54.2
O-C/SiO2	532.4	1.6	43.4
-OH	533.6	1.9	2.4

Peak assignment	Binding energy, eV	FWHM, eV	Area, %
S 2p3/2			
cNR			
CdS	161.3	1.0	92.5
C-S/ R-SH	162.9	1.4	7.5
cNR-PEG-[Co(dmgH)2(py)Cl]			
CdS	161.1	1.0	81.7
C-S/ R-SH	162.1	1.4	18.3
cNR-PEG-[Co]+ [Co]-			
CdS	161.0	1.1	65.4
C-S/ R-SH	163.0	1.2	34.6
cNR-PEG-TBA+ [Co(BPh2)2]-			
CdS	161.2	1.4	89.5
C-S/ R-SH	163.0	1.3	10.5
cNR-PEG-[Co]+ [Co(BPh2)2]-			
CdS	161.0	1.0	87.6
C-S/ R-SH	163.0	1.3	12.4
cNR-[Co]+ BArF-			
CdS	161.0	1.0	66.1
C-S/ R-SH	163.0	1.2	33.9
Peak assignment	Binding energy, eV	FWHM, eV	Area, %
Co 2p3/2			
cNR-PEG-[Co(dmgH)2(py)Cl]			
Co-complex	781.7	3.5	-
cNR-PEG-[Co]+ [Co]-			
Co-complex	781.8	3.5	-
cNR-PEG-TBA+ [Co(BPh2)2]-			
Co-complex	780.4	2.3	-
cNR-PEG-[Co]+ [Co(BPh2)2]-			
Co-complex	781.2	3.5	-
cNR-[Co]+ BArF-			
Co-complex	781.7	3.5	-

Table S 2: The different Cd and Co contents determined by TXRF, the calculated number of rods and Co centers per rod and the determined hydrogen per rod and per Co center for all five photocatalytic systems

sample	Cd content [mmol/L]	Co content [mmol/L]	Number of rods	Number of Co centers per rod	H2/rod	H2/Co center
cNR	-	-	-	-	0	0
cNR-PEG-$[Co]^{+}[Co]^{-}$ (I)	2.1±0.2	0.060±0.002	$(2.0\pm0.2)\cdot10^{12}$	356±41	7755±163	22±1
cNR-PEG-$[Co(dmgH)_2(ppy)Cl]$ (II)	2.0±0.8	0.063±0.003	$(1.9\pm0.8)\cdot10^{12}$	399±177	10607±235	27±1
cNR-PEG-$[Co]^{+}BArF^{-}$ (III)	1.9±0.1	0.11±0.01	$(1.8\pm0.1)\ 10^{12}$	718±134	8553±31	12±2
cNR-PEG-$TBA^{+}[Co(BppH_2)_2]^{-}$ (IV)	2.0±0.2	0.534±0.050	$(1.9\pm0.1)\ 10^{12}$	3389±568	18252±105	5±1
cNR-PEG-$[Co]^{+}[Co(BPPh_2)_2]^{-}$ (V)	3.6±0.1	0.588±0.112	$(3.5\pm0.1)\ 10^{12}$	2021±461	7741±79	4±1

3. Supporting figures

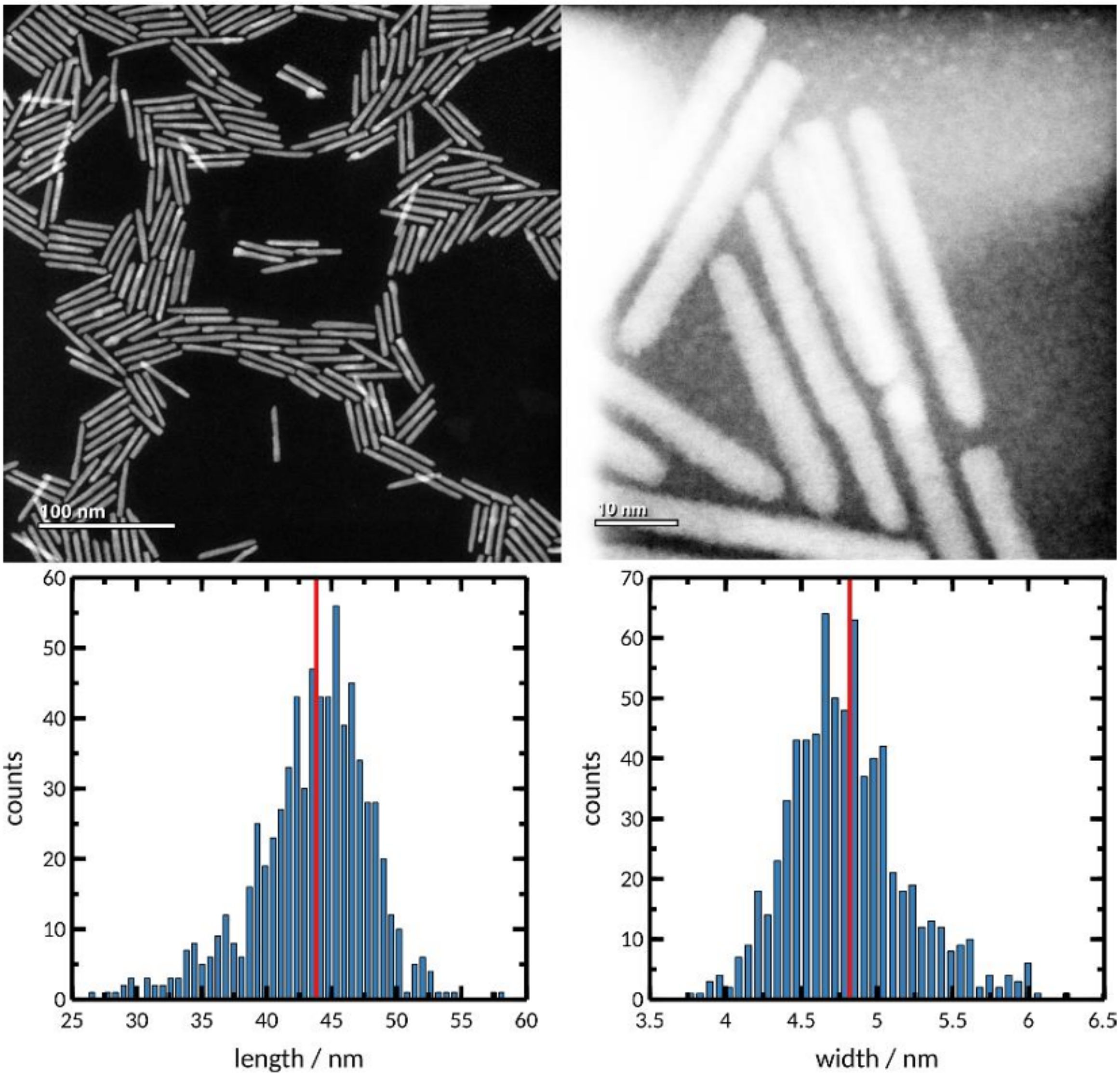

Figure S 1: Representative STEM-ADF images of the nanorods used and histograms of their length and width. 640 individual particles were analyzied, yielding an average length of 43.8 ± 5.8 nm and width of 4.8 ± 0.4 nm for the *NRs. Figure reproduced from Boecker, Marcel, et al. "Rhodium-Complex-Functionalized and Polydopamine-Coated CdSe@ CdS Nanorods for Photocatalytic NAD+ Reduction." ACS Applied Nano Materials 4.12 (2021): 12913-12919.licensed under* [CC BY 4.0]

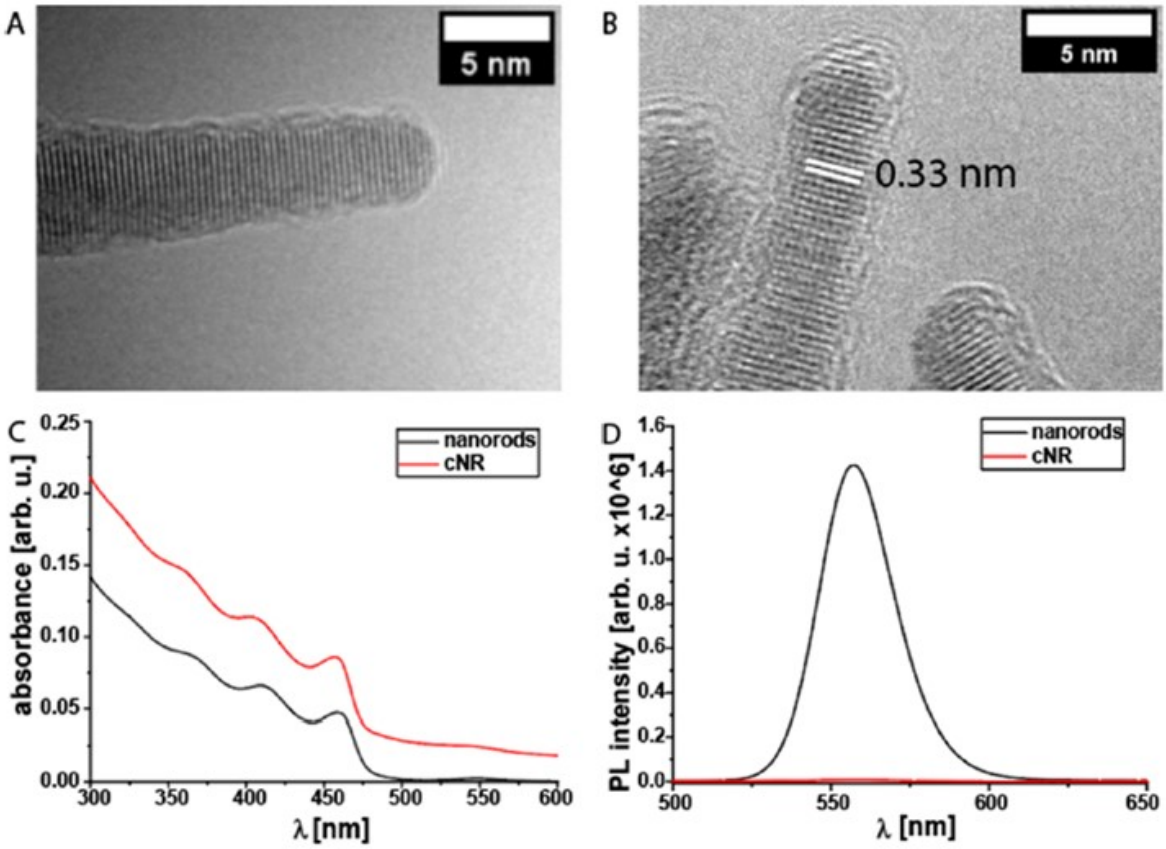

Figure S 2: (A,B) TEM images of (A) pure NRs and (B) cNRs, with the measurement of the lattice spacing (0.33 nm) for the (002) plane, which is characteristic of the CdSe@CdS NRs. (C) Absorption spectra of bare NRs and cNRs functionalized with PEG. (D) Photoluminescence spectra (λ_{ex} = 450 nm) of bare NRs and cNRs functionalized with PEG in water. Figure reproduced from Boecker, Marcel, et al. "Rhodium-Complex-Functionalized and Polydopamine-Coated CdSe@ CdS Nanorods for Photocatalytic NAD+ Reduction." ACS Applied Nano Materials 4.12 (2021): 12913-12919.licensed under CC BY 4.0

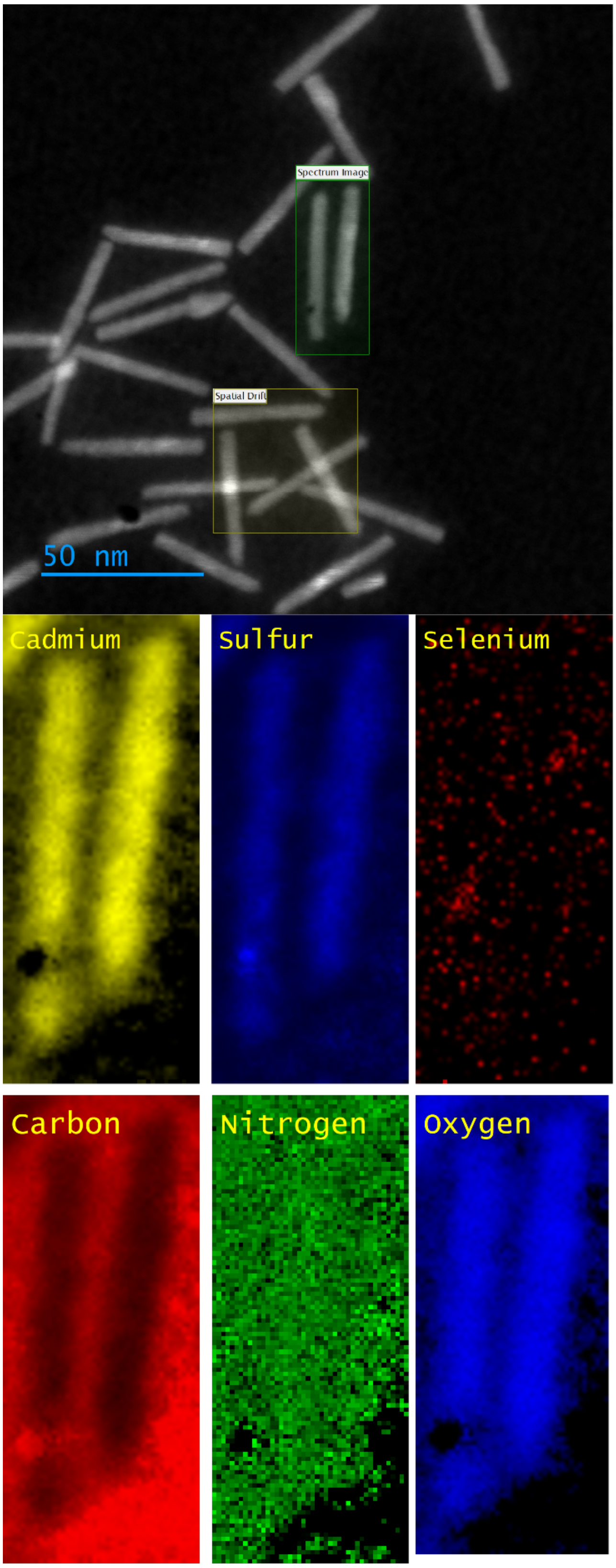

Figure S 3: EELS mapping of the NR and PDA elements for the cNR. the TEM image showing the measured nanorods (top), and the EELS mapping of the NR elements (middle) and the polymer elements (bottom).

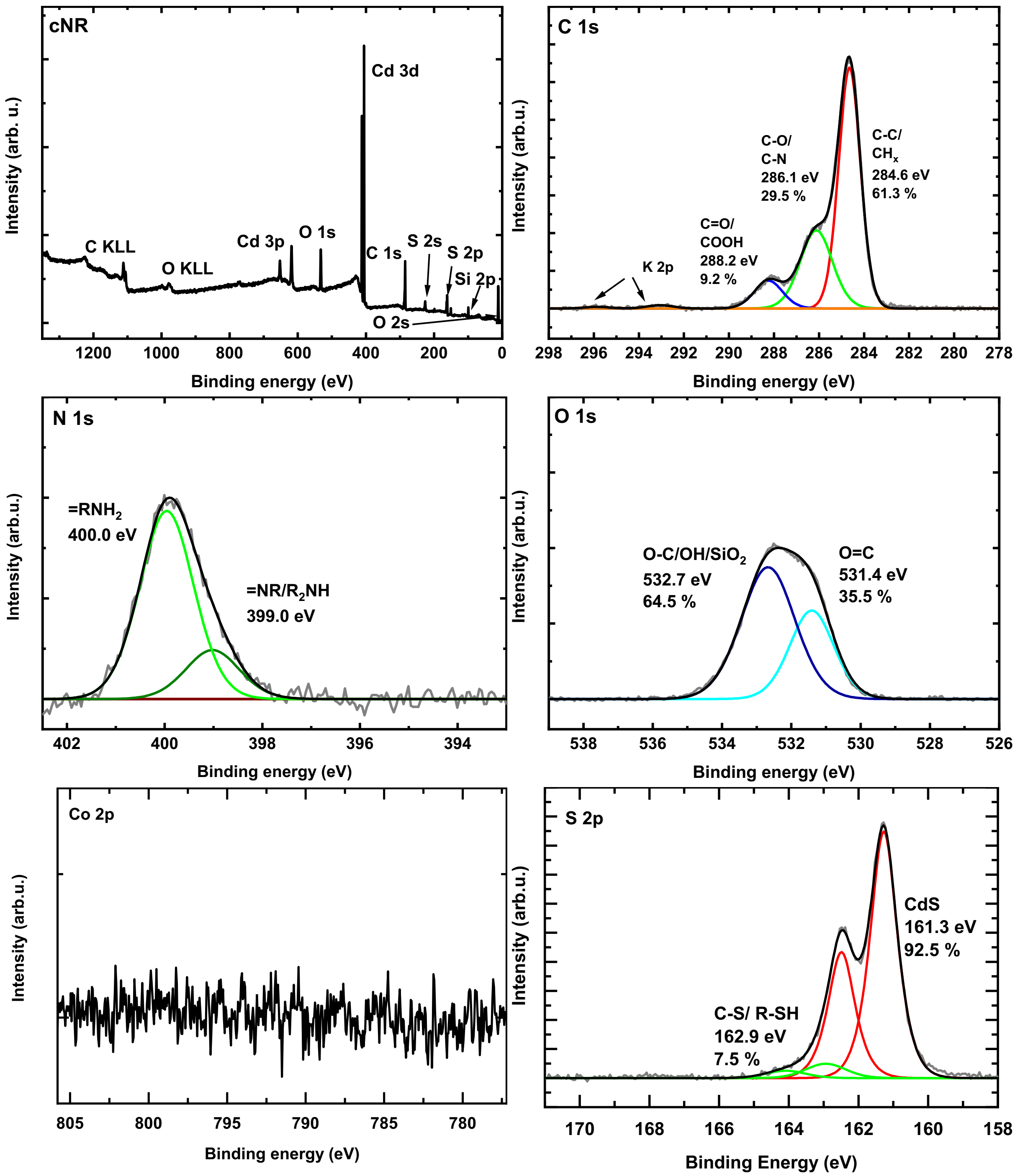

Figure S 4:XP survey spectrum (A), the high-resolution C 1s (B), N 1s (C), O 1s (D), Co 2p (E), and S 2p (F) XP spectra of the cNR.

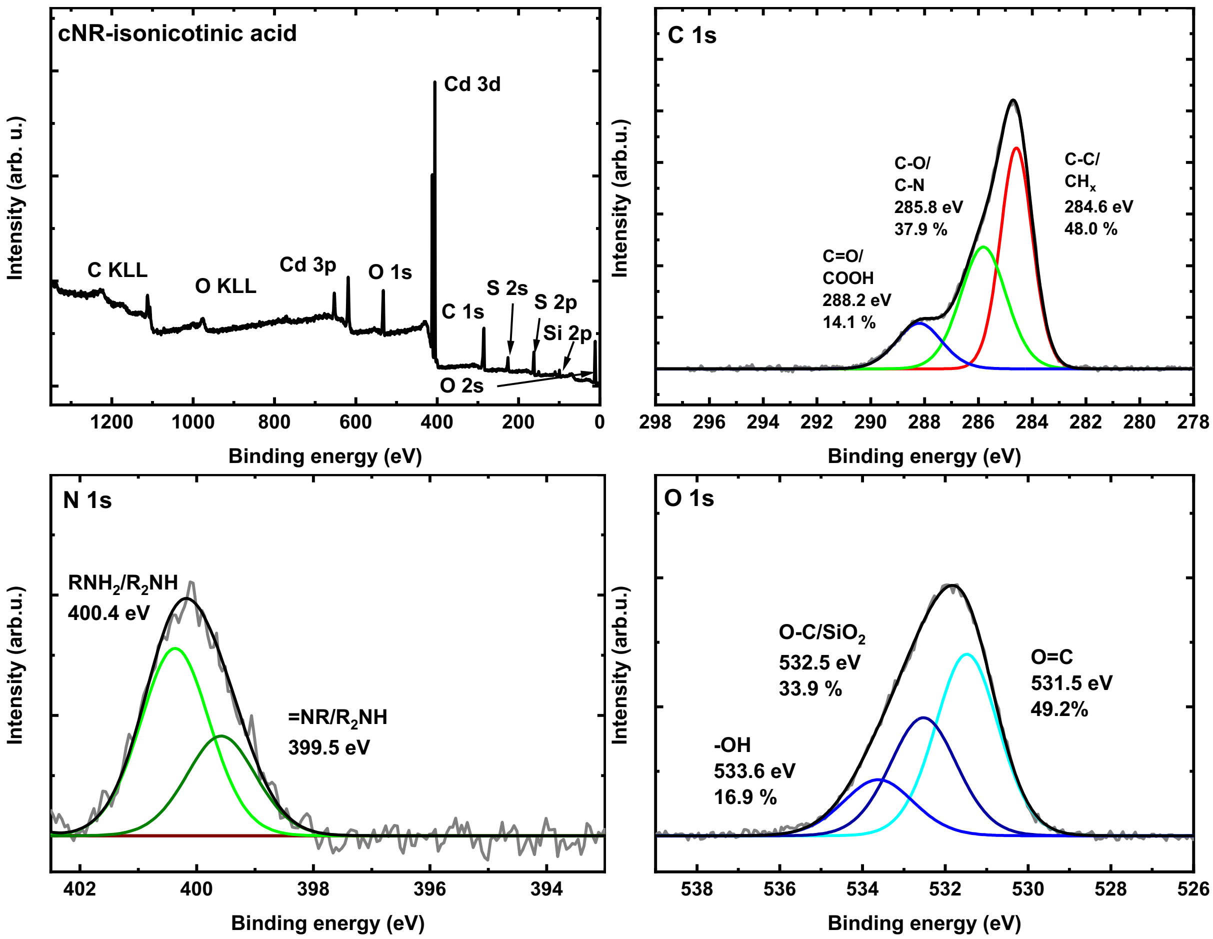

Figure S 5: XP survey spectrum (A), the high-resolution C 1s (B), N 1s (C), and O 1s (D) spectra of the cNR with isonicotinic acid functionalized.

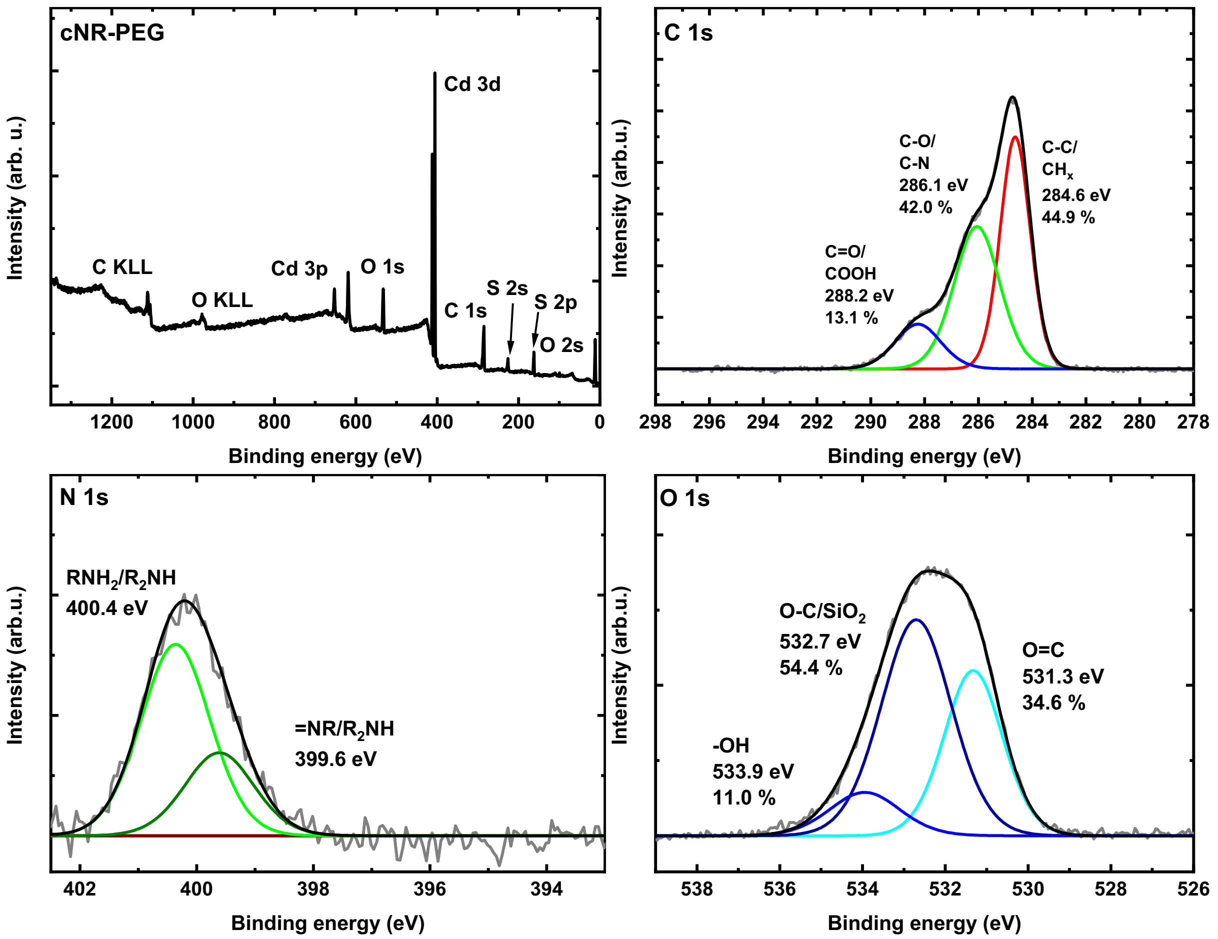

Figure S 6: XP survey spectrum (A), the high-resolution C 1s (B), N 1s (C), and O 1s (D) spectra of the cNR-PEG.

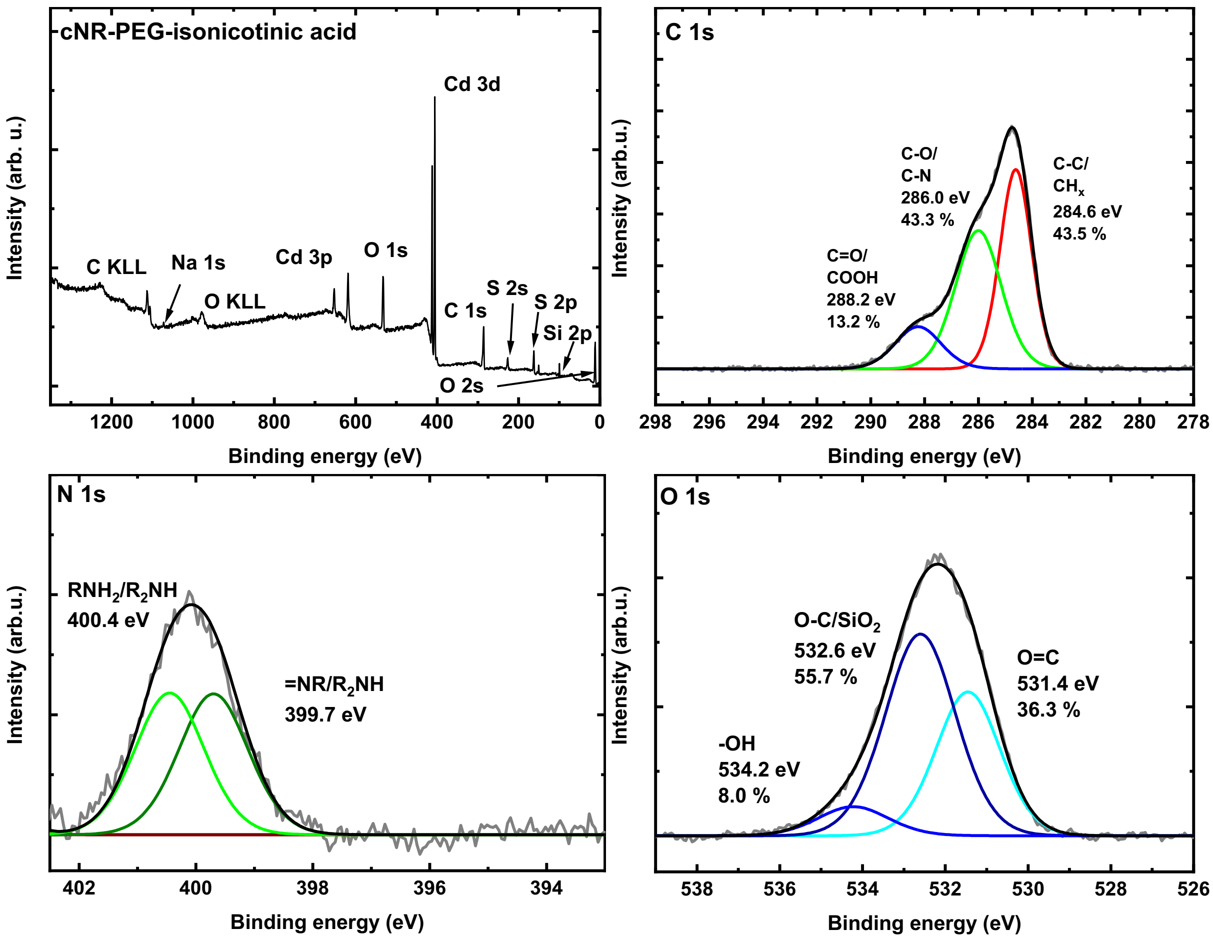

Figure S 7 XP survey spectrum (A), the high-resolution C 1s (B), N 1s (C), and O 1s (D) spectra of the cNR-PEG and isonicotinic acid.

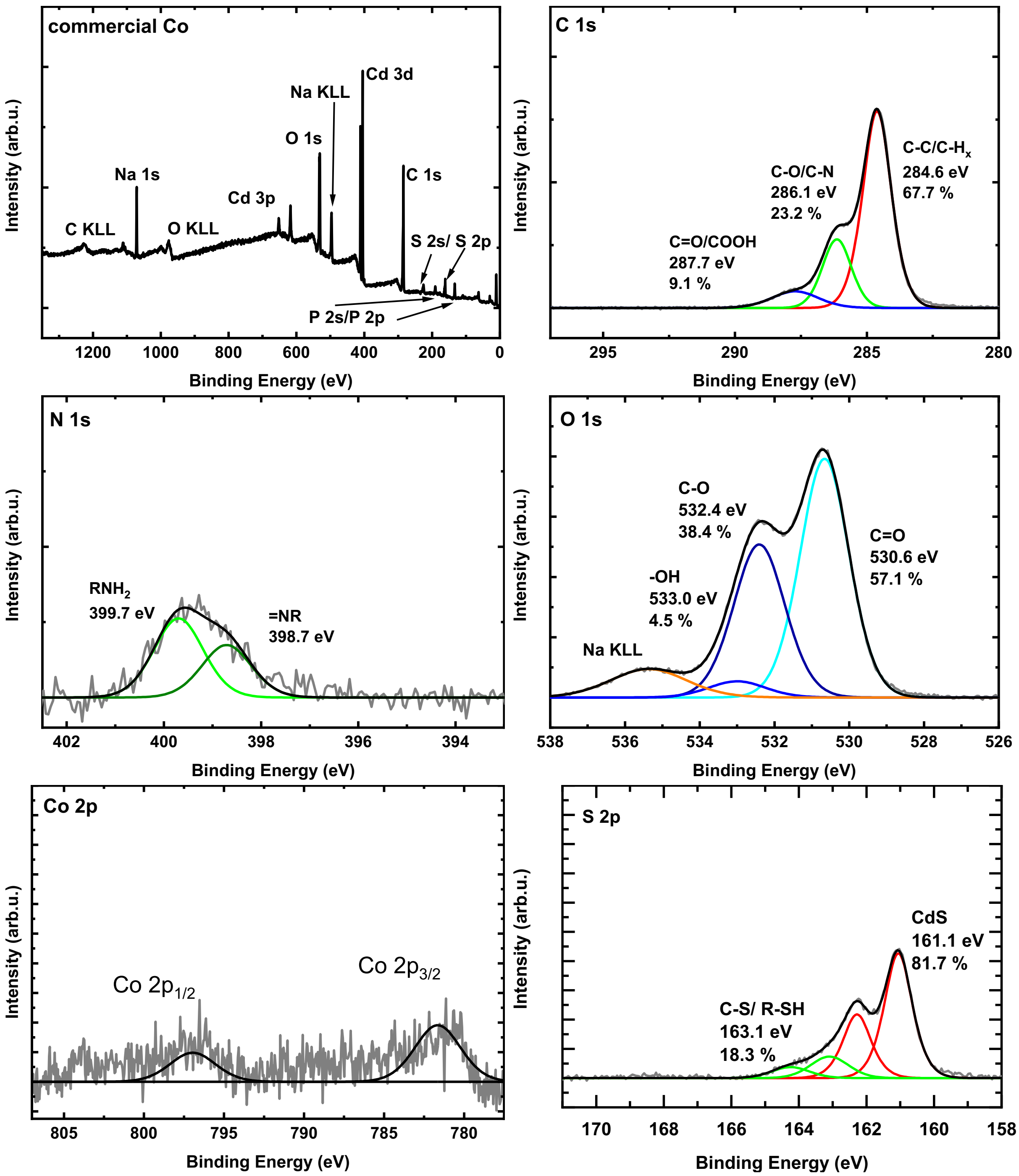

Figure S 8: XP survey spectrum (A), the high-resolution C 1s (B), N 1s (C), O 1s (D), Co 2p (E), and S 2p (F) XP spectra of the photocatalytic system cNR-PEG-[Co(dmgH)$_2$(py)Cl].

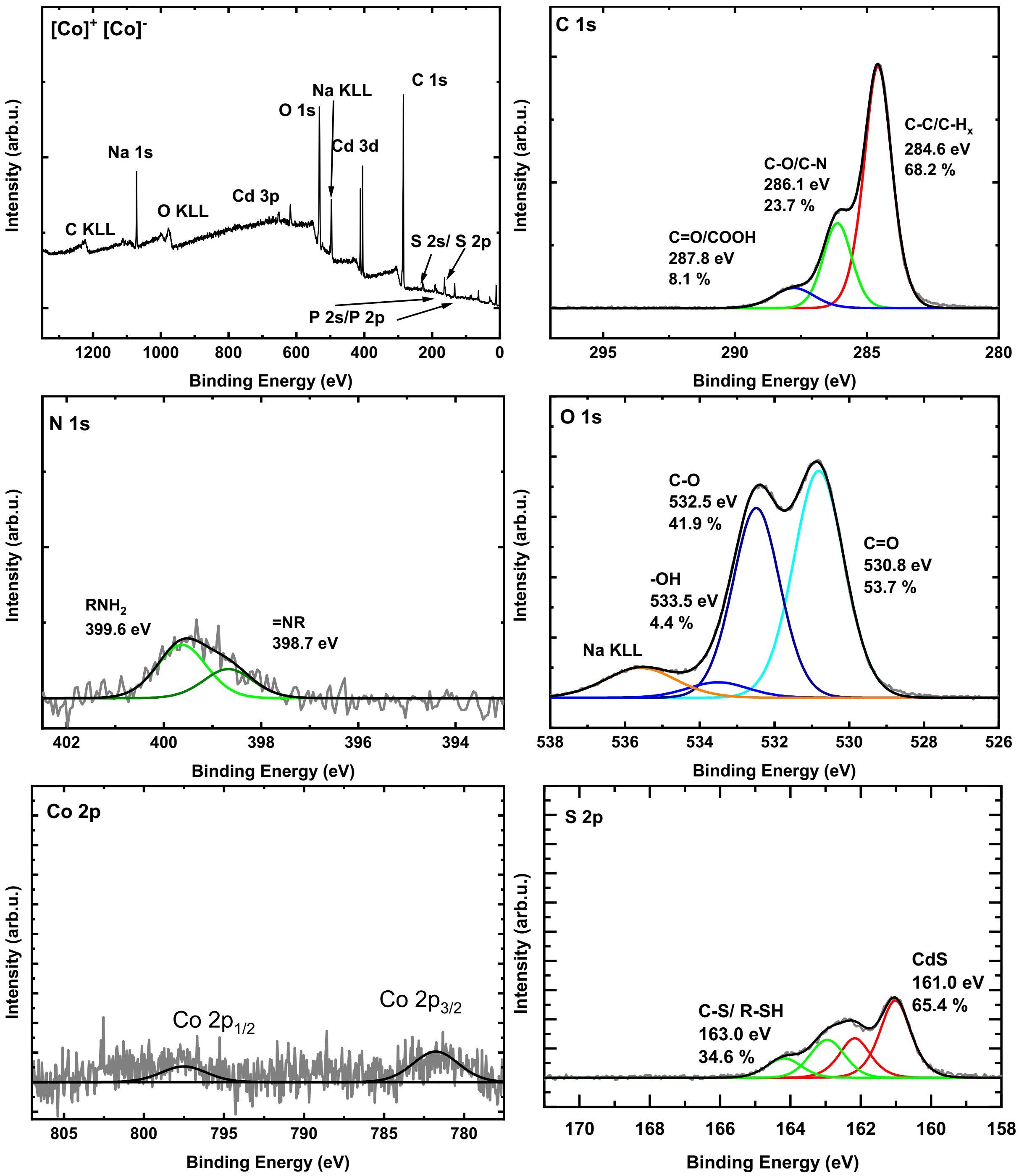

Figure S 9: XP survey spectrum (A), the high-resolution C 1s (B), N 1s (C), O 1s (D), Co 2p (E), and S 2p (F) XP spectra of the photocatalytic system cNR-PEG-[Co]⁺ [Co]⁻.

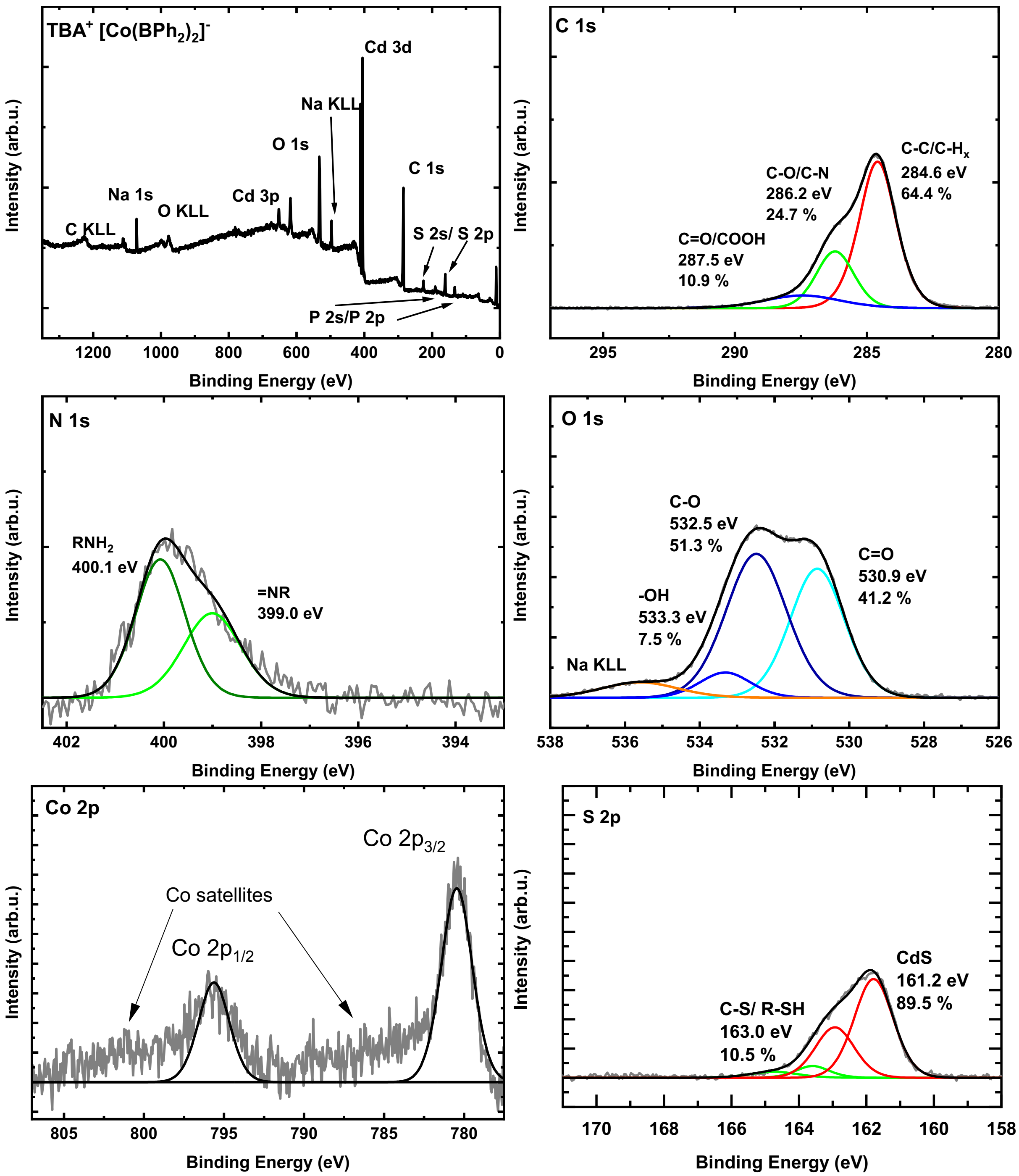

Figure S 10: XP survey spectrum (A), the high-resolution C 1s (B), N 1s (C), O 1s (D), Co 2p (E), and S 2p (F) XP spectra of the photocatalytic system cNR-PEG-TBA⁺ [Co(BPh₂)₂]⁻.

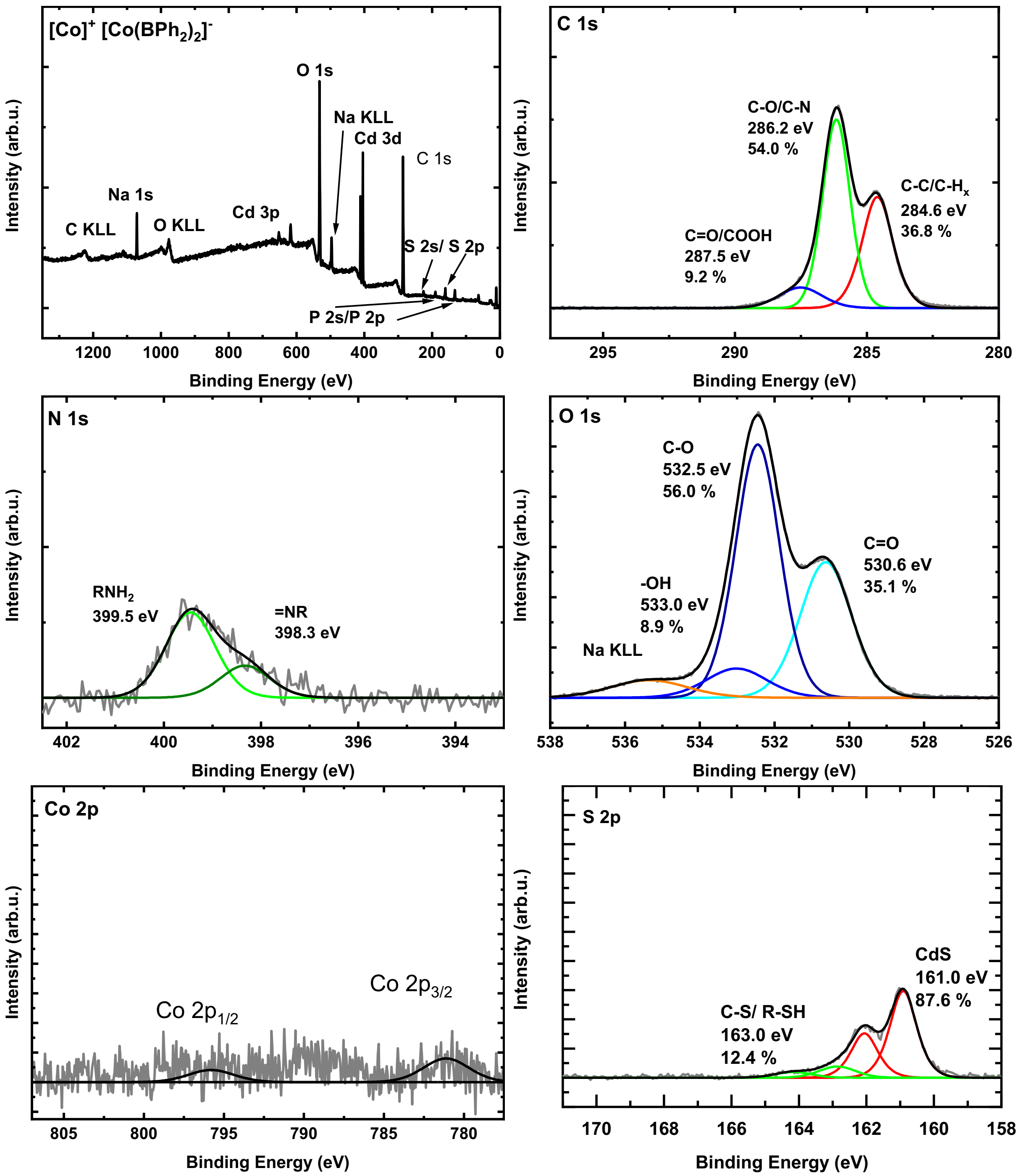

Figure S 11: XP survey spectrum (A), the high-resolution C 1s (B), N 1s (C), O 1s (D), Co 2p (E), and S 2p (F) XP spectra of the photocatalytic system cNR-PEG-$[Co]^+ [Co(BPh_2)_2]^-$.

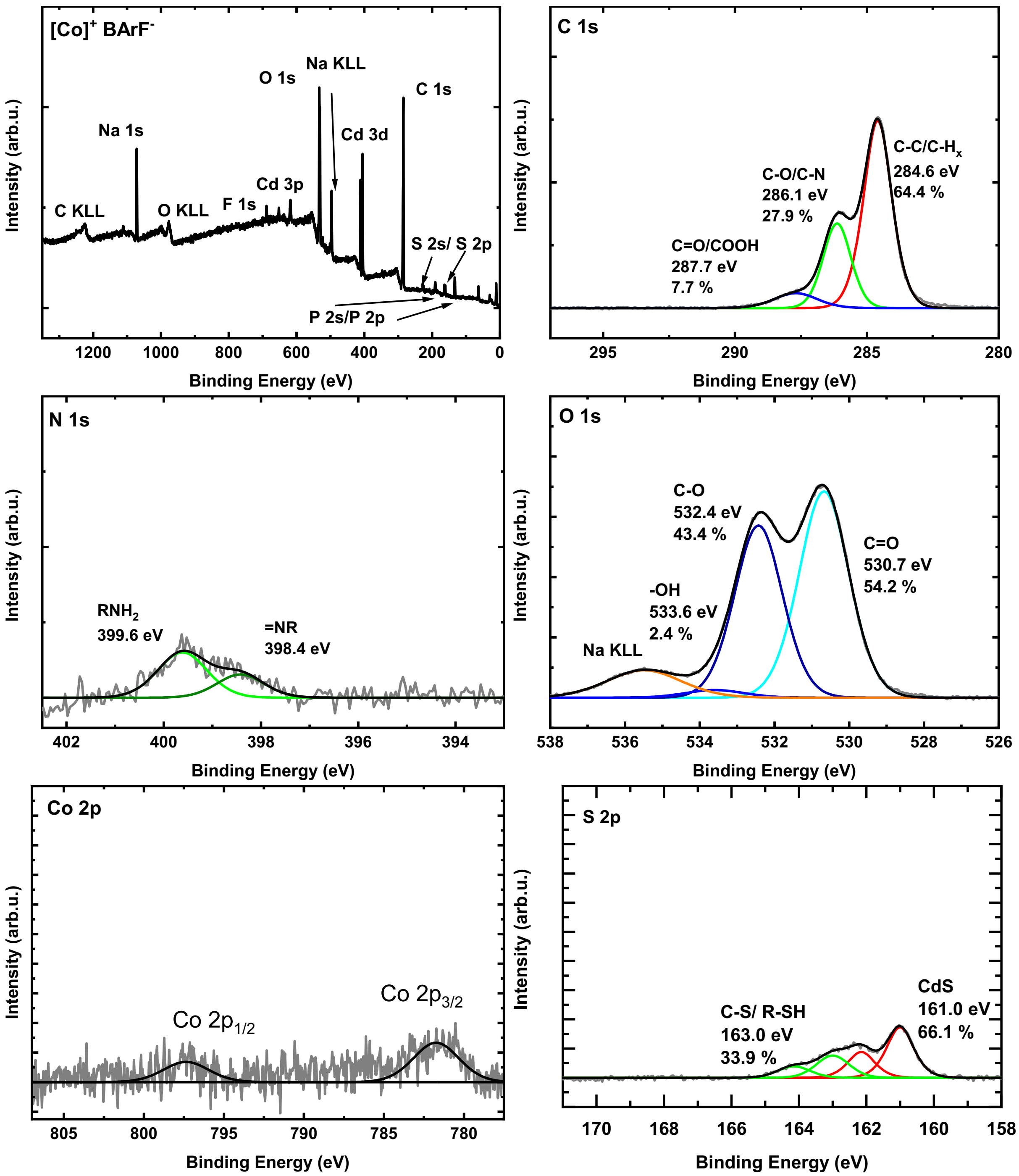

Figure S 12: XP survey spectrum (A), the high-resolution C 1s (B), N 1s (C), O 1s (D), Co 2p (E), and S 2p (F) XP spectra of the photocatalytic system cNR-[Co]$^{+}$ BArF^{-}.

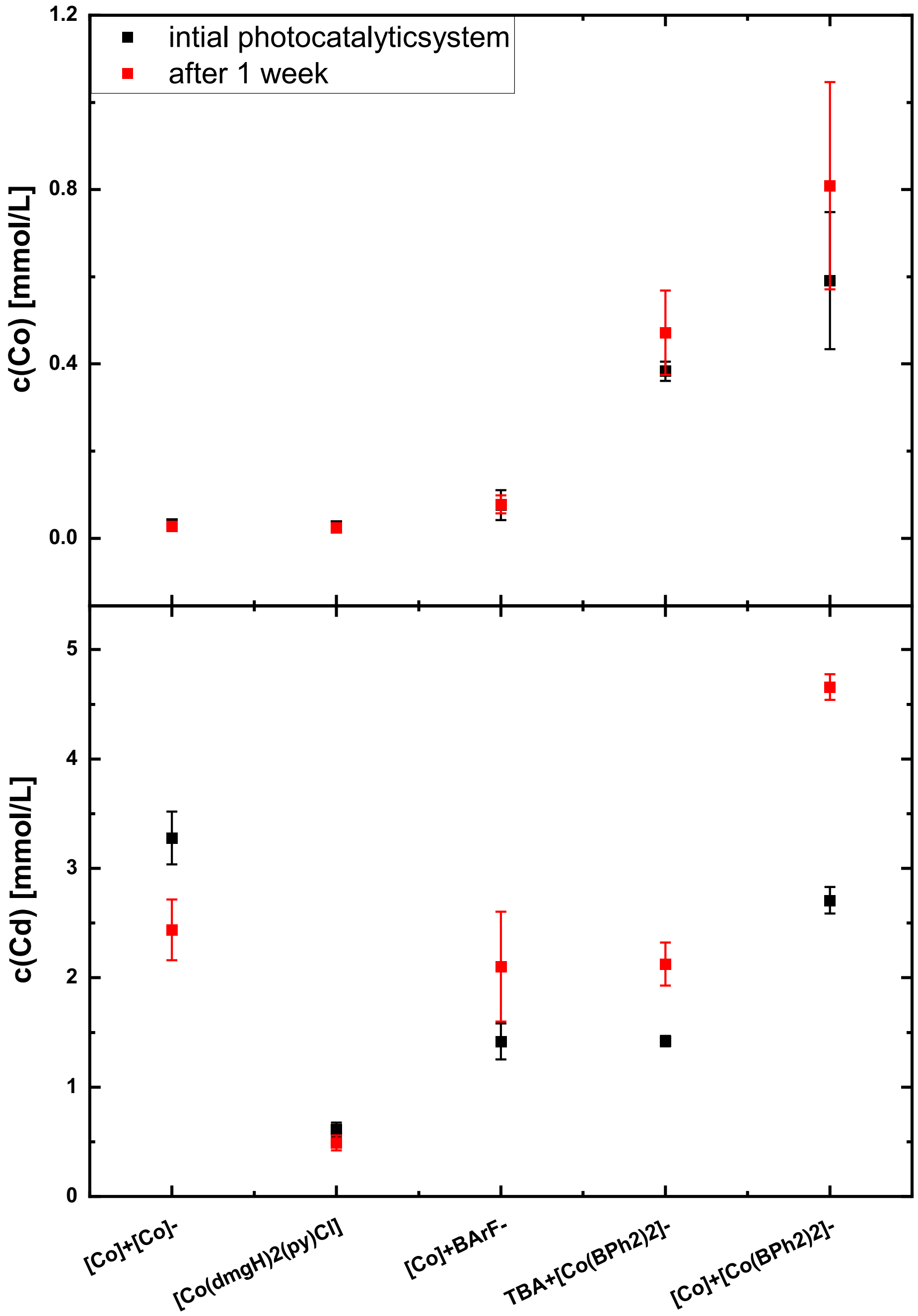

Figure S 13: TXRF results for the concentration of cobalt and cadmium of the initial photocatalytic system and after a week of storing.

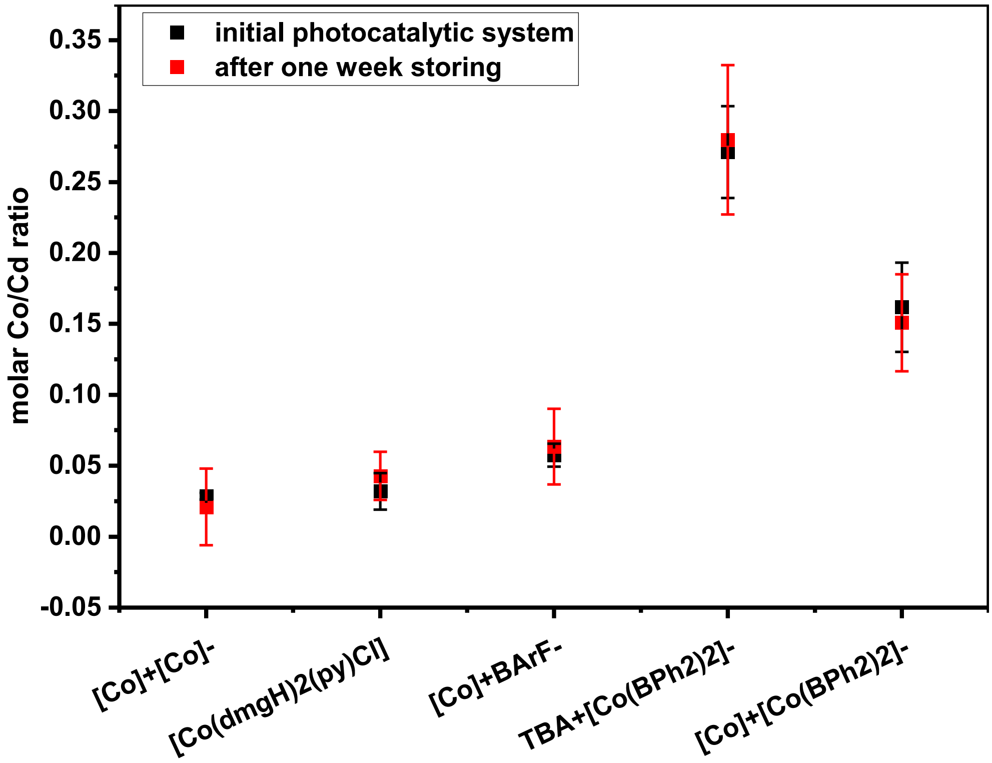

Figure S 14: Molar ratio of Co to Cd for the initial photocatalytic system and after one week of storing determined by TXRF.

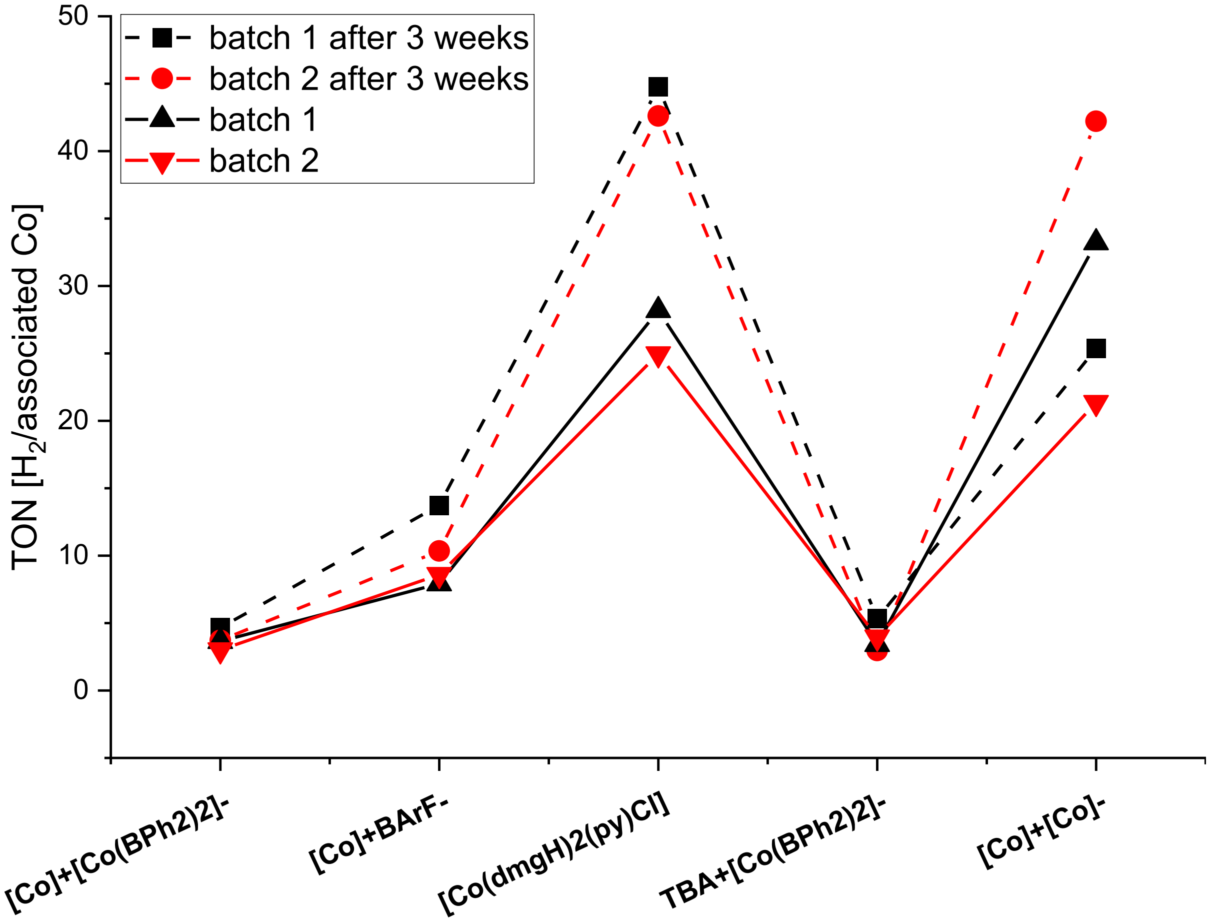

Figure S 15: Stability of hydrogen production efficiency after 3 weeks of storage. An increase in activity in some samples can be explained by solvent evaporation and hence an increase in overall concentration.

4. References

(1) Boecker, M.; Micheel, M.; Mengele, A. K.; Neumann, C.; Herberger, T.; Marchesi D'Alvise, T.; Liu, B.; Undisz, A.; Rau, S.; Turchanin, A.; Synatschke, C. V.; Wächtler, M.; Weil, T. Rhodium-Complex-Functionalized and Polydopamine-Coated CdSe@CdS Nanorods for Photocatalytic NAD+ Reduction. *ACS Appl Nano Mater* **2021**, *4* (12), 12913–12919. https://doi.org/10.1021/acsanm.1c02994.

(2) Carbone, L.; Nobile, C.; De Giorgi, M.; Della Sala, F.; Morello, G.; Pompa, P.; Hytch, M.; Snoeck, E.; Fiore, A.; Franchini, I. R.; Nadasan, M.; Silvestre, A. F.; Chiodo, L.; Kudera, S.; Cingolani, R.; Krahne, R.; Manna, L. Synthesis and Micrometer-Scale Assembly of Colloidal CdSe/CdS Nanorods Prepared by a Seeded Growth Approach. *Nano Lett* **2007**, *7* (10), 2942–2950. https://doi.org/10.1021/nl0717661.

(3) Jasieniak, J.; Smith, L.; Van Embden, J.; Mulvaney, P.; Califano, M. Re-Examination of the Size-Dependent Absorption Properties of CdSe Quantum Dots. *J. Phys. Chem. C* **2009**, *113* (45), 19468–19474. https://doi.org/10.1021/jp906827m.

(4) Talapin, D. V.; Nelson, J. H.; Shevchenko, E. V.; Aloni, S.; Sadtler, B.; Alivisatos, A. P. Seeded Growth of Highly Luminescent CdSe/CdS Nanoheterostructures with Rod and Tetrapod Morphologies. *Nano Lett* **2007**, *7* (10), 2951–2959. https://doi.org/10.1021/nl072003g.

(5) Amirav, L.; Alivisatos, A. P. Photocatalytic Hydrogen Production with Tunable Nanorod Heterostructures. *J. Phys. Chem. Lett.* **2010**, *1* (7), 1051–1054. https://doi.org/10.1021/jz100075c.

(6) Oswald, E.; Gaus, A. L.; Kund, J.; Küllmer, M.; Romer, J.; Weizenegger, S.; Ullrich, T.; Mengele, A. K.; Petermann, L.; Leiter, R.; Unwin, P. R.; Kaiser, U.; Rau, S.; Kahnt, A.; Turchanin, A.; von Delius, M.; Kranz, C. Cobaloxime Complex Salts: Synthesis, Patterning on Carbon Nanomembranes and Heterogeneous Hydrogen Evolution Studies. *Chem. Eur. J.* **2021**, *27* (68), 16896–16903. https://doi.org/10.1002/chem.202102778.

(7) Xu, Y. N., & Ching, W. Y. (1993). Electronic, optical, and structural properties of some wurtzite crystals. *Phys. Rev. B*, *48*(7), 4335.
